# Supplementary material for: Reconstitution of microtubule into GTP-responsive nanocapsules
Source: Nat Commun. 2022 Sep 15;13:5424. doi: 10.1038/s41467-022-33156-5 (PMC9477877; doi:10.1038/s41467-022-33156-5)
Supplement: Supplementary file 1 — Supplementary Information [file 41467_2022_33156_MOESM1_ESM.pdf]

Supplementary Information for

Reconstitution of microtubule into GTP-responsive nanocapsules

Authors: Noriyuki Uchida[1,2], Ai Kohata[3], Kou Okuro[4,5], Annalisa Cardellini[6], Chiara Lionello[6], Eric A. Zizzi[7], Marco A. Deriu[7], Giovanni M. Pavan[6,8], Michio Tomishige[9], Takaaki Hikima[10], and Takuzo Aida[1,3]*

Affiliations:

[1] RIKEN Center for Emergent Matter Science, 2-1 Hirosawa, Wako, Saitama 351-0198, Japan.

[2] Department of Applied Chemistry, Graduate School of Engineering, Tokyo University of Agriculture and Technology, 2-24-16 Naka-cho, Koganei, Tokyo 184-8588, Japan.

[3] Department of Chemistry and Biotechnology, School of Engineering, The University of Tokyo, 7-3-1 Hongo, Bunkyo-ku, Tokyo 113-8656, Japan.

[4] Department of Chemistry, The University of Hong Kong, Pokfulam Road, Hong Kong, China.

[5] State Key Laboratory of Synthetic Chemistry, The University of Hong Kong, Pokfulam Road, Hong Kong, China.

[6] Department of Applied Science and Technology, Politecnico di Torino, Corso Duca degli Abruzzi 24, 10129 Torino, Italy.

[7] PolitoBIOMedLab, Department of Mechanical and Aerospace Engineering, Politecnico di Torino, Corso Duca degli Abruzzi 24, 10129 Torino, Italy.

[8] Department of Innovative Technologies, University of Applied Sciences and Arts of Southern Switzerland, Polo Universitario Lugano, Campus Est, Via la Santa 1, 6962 Lugano-Viganello, Switzerland.

[9] Department of Physical Sciences, Aoyama Gakuin University, Kanagawa 252-5258, Japan.

[10] RIKEN SPring-8 Center, 1-1-1 Kouto, Sayo, Hyogo 679–5198 Japan.

*Correspondence to: aida@macro.t.u-tokyo.ac.jp (T.A.)

Table of contents

1. **Supplementary Methods** S3
2. **Supplementary Notes**.. S32
3. **Supplementary References** S61

1. Supplementary Methods

1-1. Materials

Unless indicated otherwise, all commercial reagents were used as received. Bovine serum albumin (BSA), doxorubicin hydrochloride (DOX), uridine-5'-triphosphate trisodium salt (UTP), cytidine-5'-triphosphate disodium salt (CTP), guanosine-5'-triphosphate trisodium salt (GTP), guanosine-5'-diphosphate sodium salt (GDP), adenosine-5'-triphosphate disodium salt (ATP), and Dulbecco's phosphate-buffered saline (D-PBS) were purchased from Wako Pure Chemical Industries. Fluorescein isothiocyanate (FITC) and 1,4-piperazinediethanesulfonic acid (PIPES) were purchased from Tokyo Chemical Industry (TCI). Guanylyl 5'-α, β-methylenediphosphonate (GTP*) was purchased from Cosmobio. Foetal bovine serum (FBS) was purchased from Thermo Fisher Scientific. Eagle's minimal essential medium (EMEM) was purchased from Life Technologies. Polyacrylamide gel was purchased from Bio-Rad. Biomol Green™ Reagent was purchased from Enzo Life Sciences. Cell Counting Kit-8 was purchased from Dojindo Laboratories. The human hepatocellular carcinoma Hep3B cell line (ATCC® HB-8064™) was purchased from American Type Culture Collection (ATCC). Human lung carcinoma A547 cell was provided by the RIKEN BRC. HeLa cell was purchased from JCRB Cell Bank. α-Mercaptoethyl-ω-methoxy, polyoxyethylene (PEG-SH, M_w; 5 K) was purchased from NOF. Gold nanoparticle (50 nm in diameter) and guanosine 5'-O-(3-thiotriphosphate (GTPγS) were purchased from Sigma–Aldrich. Polyoxyethylene sorbitan monolaurate (Tween 20) was purchased from Nacalai Tesque.

1-2. General procedures

^{1}H NMR and ^{13}C NMR spectra were recorded on a JEOL model GSX-500 spectrometer, where chemical shifts (δ in ppm) were determined using $CHCl_3$ (δ 7.26), $CHD_2(CD_3)SO$ (δ 2.50), and HDO (δ 4.79) for ^{1}H NMR and $CDCl_3$ (δ 77.2) and $(CD_3)_2SO$ (δ 39.5) for ^{13}C NMR as internal

references. Matrix-assisted laser desorption ionization time-of-flight (MALDI-TOF) mass spectrometry was performed with *a*-cyano-4-hydroxycinnamic acid (CCA), 2,5-dihydroxybenzoic acid (DHB), or sinapinic acid (SA) as a matrix and an Applied Biosystems Biospectrometry Workstation™ model Voyager-DE™ STR spectrometer or a Bruker model Autoflex Speed™ spectrometer. A Horiba model AS-212 compact pH meter was used for pH measurements. Electronic absorption spectra were recorded with a Molecular Devices model SpectraMax Paradigm multimode microplate detection platform, a Thermo Scientific model NanoDrop 2000c spectrophotometer, or a JASCO Model V-570 spectrophotometer. Fluorescence spectra were recorded using a Molecular Devices model SpectraMax Paradigm multimode microplate detection platform. UV exposure for photoreaction was performed with a UVP model UVLM-28 UV lamp. Analyses of images of the acrylamide gels were performed using an Amersham Biosciences model Typhoon 9410 variable image analyser. Recycling preparative gel permeation chromatography (GPC) was performed with a Japan Analytical Industry model LC908-C60 using a column set consisting of JAIGEL 1H-40 and 2H-40. Transmission electron microscopy (TEM) images were recorded using a JEOL model JEM-1400 electron microscope operating at an anode voltage of 120 kV. Samples were applied to an electron microscope specimen grid covered with a thin carbon support film that had been hydrophilized by ion bombardment. Then, the samples were negatively stained with a saturated uranyl acetate solution. Tapping-mode atomic force microscopy (AFM) of air-dried samples on a mica surface was performed using an SII Nano Technology model NanoNavi S-image. Small-angle X-ray scattering (SAXS) was carried out at BL45XU in SPring-8 (Hyogo, Japan)[S1] with a Dectris model Pilatus 3X 300K-W detector. Scattering vector q ($q = 4\pi\sin\theta/\lambda$; 2θ and λ are the scattering angle and wavelength of the incident X-ray beam [1.70 Å], respectively) and the position of an incident X-ray beam on the detector were calibrated using several orders of layer reflections from silver behenate (d = 58.380 Å). Dynamic light scattering (DLS) measurements were performed with a Malvern model Zetasizer μV light scattering

spectrometer using an infrared laser (830 nm). Zeta potential measurements were performed using a Malvern model Zetasizer Nano ZSP zeta potential analyser. Confocal laser scanning microscopy (CLSM) was performed using a Leica model TCS SP8 microscope. Flow cytometry was performed using a BD model Accuri® C6 flow cytometer with BD Accuri C6 software in which the thresholds were set at 80,000 forward scatter (FSC) and at 0 side scatter (SSC) using detectors FL1 for green fluorescence and FL3 for red fluorescence to distinguish positive and negative sained cells . Asymmetric field flow fractionation was performed using an Eclipse model AF4 separation system equipped with a JASCO model UV-2070$_{plus}$ variable-wavelength UV-Vis detector. A regenerated cellulose membrane with a 5 kD cut-off (Wyatt Technology) and 350 μm spacer to adjust the channel thickness was used in combination with PIPES buffer (10 mM PIPES and 1 mM $MgCl_2$, pH 6.8) as an eluent (detector-flow rate: 1.0 ml min^{-1} cross-flow rate: gradient from 0.5 ml min^{-1} to 0 ml min^{-1} for 180 min, transition time: 5 min). BSA was used as a standard for evaluating the concentrations of tubulin heterodimer (THD). Ultrafiltration was performed using cellulose-made centrifugal filters (Amicon® Ultra-0.5 ml 100K) with a molecular weight cut-off of 100 kDa. Concentrations of FITC molecules and gold nanoparticles were measured by determining the absorbance at 490 nm and 540 nm, respectively. All-atom molecular dynamics (MD) simulations[S2] were performed in which all molecular systems were created using AmberTools 20[S3] and simulated with the GROMACS 2020.5 package[S4], and all images were created with the Visual Molecular Dynamics (VMD) package[S5].

1-3. Synthesis of compounds

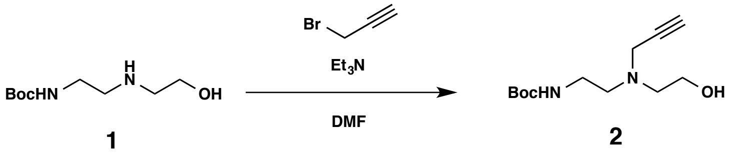

Compound **2.** To a dimethylformamide (DMF, 350 ml) solution of a mixture of **1** (15.9 g, 77.6 mmol) and Et_3N (33.2 ml, 233 mmol) was added propargyl bromide (27.7 g, 233 mmol), and the mixture was stirred for 22 h at room temperature. Then, saturated aqueous NH_4Cl (50 ml) was slowly added to the reaction mixture, and the resultant mixture was evaporated to dryness under reduced pressure. The residue was extracted with AcOEt (100 ml) and washed successively with saturated aqueous NH_4Cl (50 ml × 2) followed by brine (50 ml). An organic extract separated was evaporated to dryness under reduced pressure, and the residue was chromatographed on silica gel with AcOEt/hexane (1/1 to 4/1) as an eluent to allow isolation of **2** as yellow oil in 54% yield (10.2 g).

^{1}H NMR (500 MHz, $CDCl_3$): δ 1.45 (s, 9H), 2.21 (t, J = 2.4 Hz, 1H), 2.67−2.75 (m, 4H), 3.22 (dt, J = 5.7 Hz, 5.6 Hz, 2H), 3.46 (d, J = 2.4 Hz, 2H), 3.62 (t, J = 5.3 Hz, 2H), 4.85 (s, 1H). ^{13}C NMR (125 MHz, $CDCl_3$): δ 28.4, 38.2, 42.0, 53.1, 55.2, 58.8, 73.2, 78.2, 79.4, 156.1.

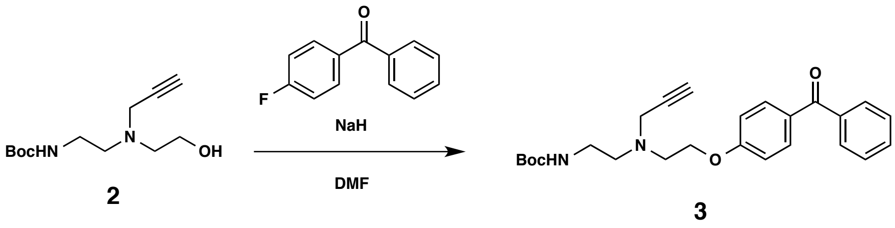

Compound **3.** To a DMF (350 ml) solution of a mixture of **2** (8.18 g 33.8 mmol) and 4-fluorobenzophenone (10.1 g, 50.6 mmol) was added sodium hydride (60% in paraffin; 2.03 g, 50.8 mmol), and the mixture was stirred for 5 h at room temperature. Then, saturated aqueous NH_4Cl (50 ml) was slowly added to the reaction mixture, and the resultant mixture was evaporated to dryness under reduced pressure. The residue was extracted with Et_2O (100 ml) and washed successively with saturated aqueous NH_4Cl (50 ml × 2) followed by brine (50 ml). An organic extract separated was evaporated to dryness under reduced pressure, and the residue was chromatographed on silica gel with AcOEt/hexane (1/5 to 1/2) as an eluent to allow isolation of **3** as yellow solid in 69% yield (9.89 g).

^{1}H NMR (500 MHz, $CDCl_3$): δ 1.35 (s, 9H), 2.16 (s, 1H), 2.66 (t, J = 3.1 Hz, 2H), 2.90 (t, J = 5.3 Hz, 2H), 3.16 (m, 2H), 3.41 (s, 2H), 4.03 (t, J = 5.3 Hz, 2H), 5.14 (br, 1H), 6.88−6.91 (m, 2H), 7.34−7.50 (m, 3H), 7.64−7.74 (m, 4H). ^{13}C NMR (125 MHz, $CDCl_3$): δ 28.4, 37.8, 42.6, 51.4, 52.9, 66.2, 73.2, 78.3, 78.9, 113.9, 128.0, 129.6, 130.0, 131.8, 132.4, 138.0, 155.9, 162.2, 195.3. MALDI-TOF-MS (*m/z*): calcd. for $C_{25}H_{30}N_2O_4K$ [M + K^+], 461.18; found, 461.05.

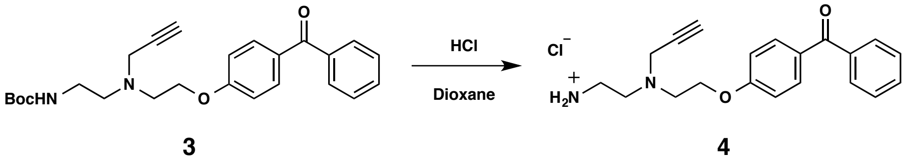

Compound **4.** A 1,4-dioxane (50 ml) solution of HCl (4 M) was added to **3** (9.45 g 22.4 mmol), and the mixture was stirred for 2 h at room temperature. Then, hexane (300 ml) was added to the reaction mixture, and the resultant suspension was filtered. The insoluble fraction, obtained by filtration, was dissolved in methanol and reprecipitated with Et_2O to allow isolation of **4** as yellow solid, quantitatively (8.22 g).

^{1}H NMR (500 MHz, D_2O): δ 3.07 (s, 1H; CCH), 3.45 (br, 2H; C$H_2NH_3^+$), 3.63−3.64 (br, 4H;), 4.06 (s, 2H), 4.15 (s, 2H), 6.67−6.68 (br, 2H), 6.97−7.19 (br, 7H). ^{13}C NMR (125 MHz, D_2O): δ 34.7, 44.0, 51.1, 53.5, 62.8, 71.3, 82.3, 115.0, 128.9, 130.3, 130.5, 133.3, 133.3, 137.5, 161.7, 197.8. MALDI-TOF-MS (*m/z*): calcd. for $C_{20}H_{21}N_2O_2K$ [M − HCl + K^+], 361.13; found, 363.05.

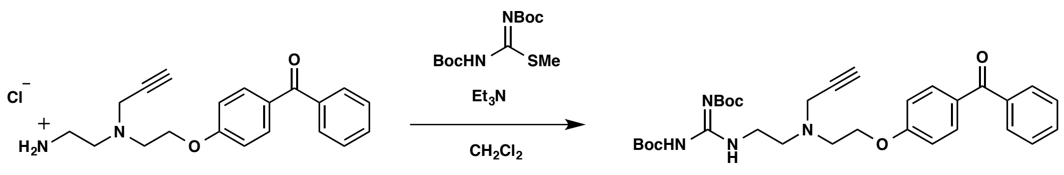

Compound **5.** To a CH_2Cl_2 (180 ml) solution of a mixture of **4** (6.50 g, 18.1 mmol) and Et_3N (12.5 ml, 90.5 mmol) was added 1,3-bis(*tert*-butoxy-carbonyl)-2-methyl-2-thiopseudo-urea (6.85 g, 23.6 mmol), and the mixture was stirred for 18 h at room temperature. Then, saturated aqueous NH_4Cl (30 ml) was slowly added to the reaction mixture, and the resultant mixture was evaporated to dryness under reduced pressure. The residue was extracted with AcOEt (60 ml) and washed with saturated aqueous NH_4Cl (30 ml × 2) followed by brine (30 ml). An organic extract separated was evaporated to dryness under reduced pressure, and the residue was chromatographed on silica gel with AcOEt/hexane (1/6 to 1/2) as an eluent to allow isolation of **5** as yellow solid in 74% yield (7.59 g).

^{1}H NMR (500 MHz, $CDCl_3$): δ 1.44−1.50 (s, 18H), 2.27 (s, 1H), 2.85 (t, J = 5.1 Hz, 2H), 3.02 (t, J = 5.3 Hz, 2H), 3.54 (m, 4H), 4.17 (t, J = 5.3 Hz, 2H), 6.96−6.99 (m, 2H), 7.43−7.58 (m, 3H), 7.73−7.81 (m, 4H), 8.67 (s, 1H), 11.48 (br, 1H). ^{13}C NMR (125 MHz, $CDCl_3$): δ 27.7, 28.1, 38.3, 42.5, 51.9, 51.9, 66.8, 73.2, 76.7, 77.0, 77.3, 78.0, 78.9, 82.5, 113.8, 127.9, 128.0, 128.0, 129.2, 129.4, 129.8, 131.6, 132.0, 132.2, 138.0, 152.6, 155.8, 162.2, 163.3, 195.1. MALDI-TOF-MS (m/z): calcd. for $C_{31}H_{40}N_4O_6K$ [M + K^+], 361.13; found, 602.91.

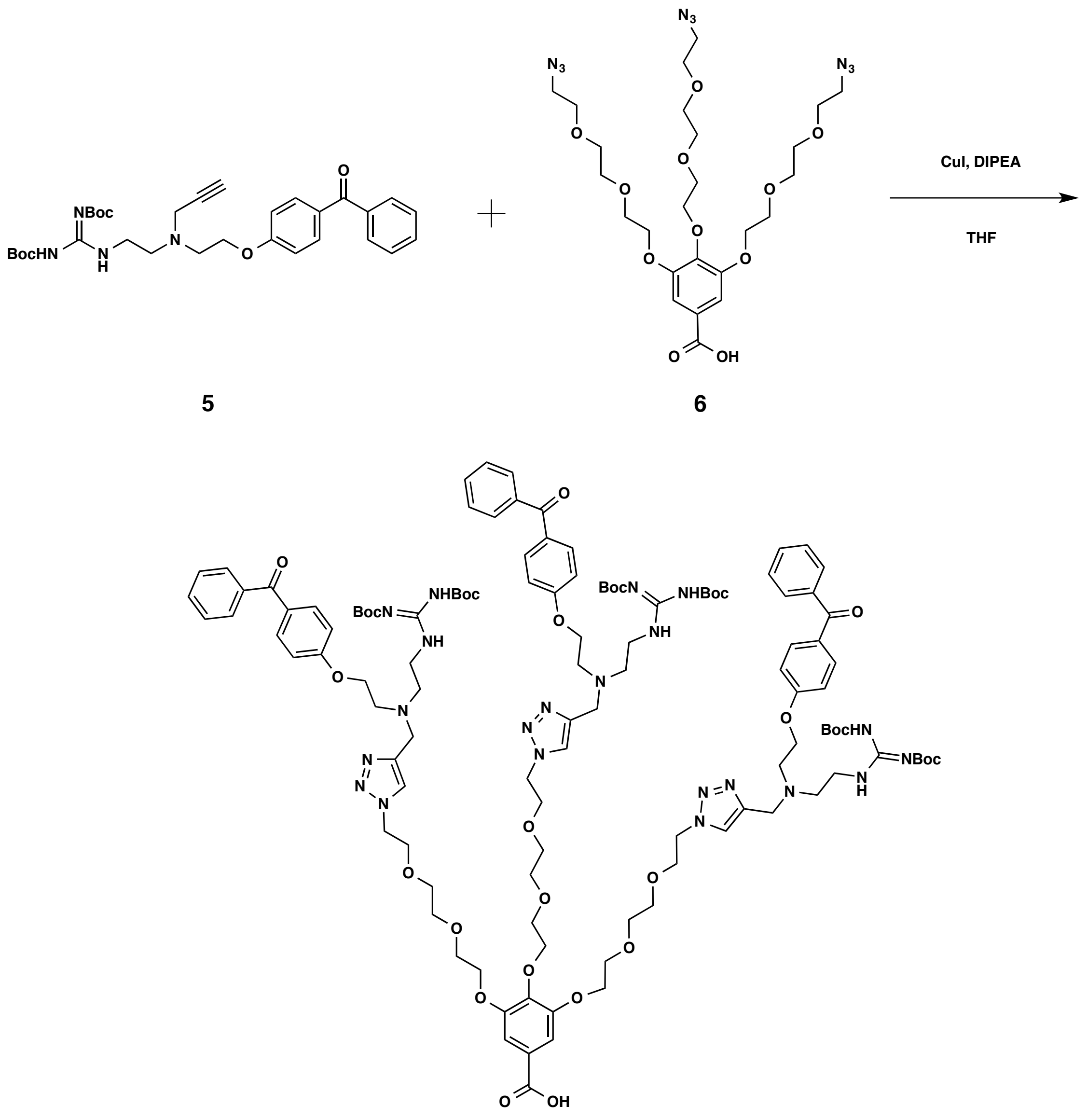

5 **6**

7

Compound **7.** To a Tetrahydrofuran (THF, 40 ml) solution of a mixture of **5** (1.28 g, 2.13 mmol), **6** (340 mg, 0.53 mmol), and diisopropylethylamine (DIPEA, 0.47 ml, 4.25 mmol) was added copper iodide (405 mg, 2.13 mmol), and the mixture was stirred for 15 h at room temperature. Then, the reaction mixture was diluted with AcOEt (50 ml) and washed with saturated aqueous NH_4Cl (30 ml × 3) followed by brine (30 ml). An organic extract separated was dried over Na_2SO_4 and filtered off from an insoluble fraction. The filtrate was subjected to recycling preparative GPC twice to allow isolation of **7** as pale green solid in 68% yield (841 mg).

^{1}H NMR (500 MHz, $CDCl_3$): δ 1.42−1.46 (s, 54H), 2.79−2.96 (br, 12H), 3.54 (br, 6H), 3.74−3.92 (br, 36H), 4.12 (br, 6H), 4.44 (br, 6H), 6.89−6.91 (br, 6H), 7.43−7.72 (br, 26H), 8.70 (s, 3H), 11.44 (br, 3H). ^{13}C NMR (125 MHz, $CDCl_3$): δ 27.5, 27.8, 38.1, 48.9, 49.7, 51.6, 66.5, 68.9, 69.9, 71.2, 78.5, 82.2, 113.4, 123.6, 127.5, 129.0, 129.3, 131.3, 131.8, 137.5, 152.2, 161.7, 162.7, 194.6. MALDI-TOF-MS (*m/z*): calcd. for $C_{95}H_{105}N_{21}O_{17}$ [M − 6Boc + H^+], 1734.85; found, 1735.04.

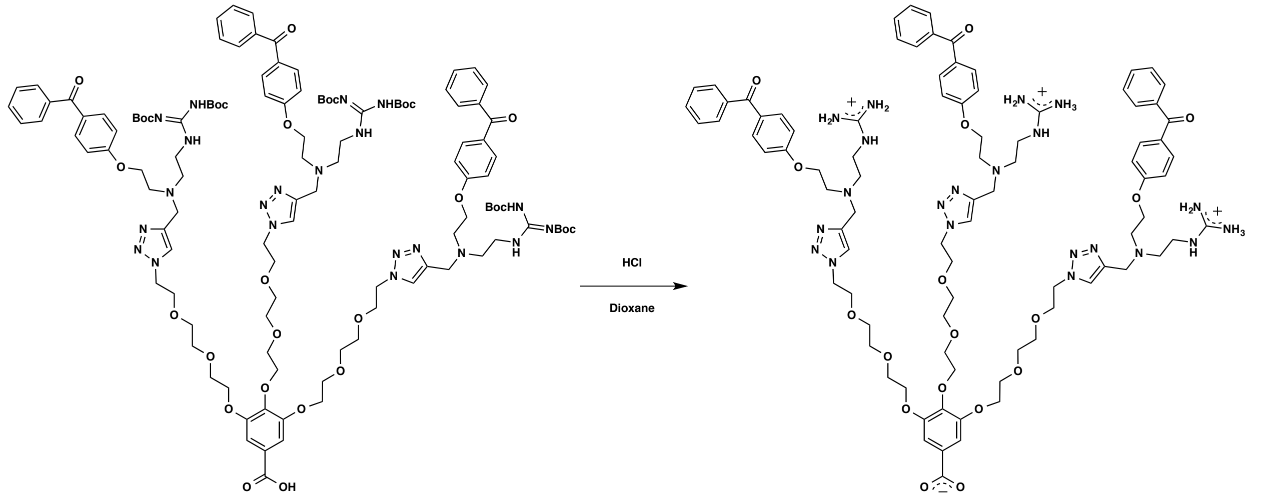

7 Glue$^{CO_2-}$

Glue$^{CO_2-}$: To a 1,4-dioxane (10 ml) solution of HCl (4 M) was added **7** (99 mg, 42 μmol)[S6], and the mixture was stirred for 4 h at room temperature. Hexane (50 ml) was added to the reaction mixture, and the mixture was filtered. The insoluble fraction was dissolved in water and then reprecipitated with 1,4-dioxane to enable the isolation of Glue$^{CO_2-}$ as a yellow solid at a 74% yield (58 mg).

^{1}H NMR (500 MHz, DMSO-*d*$_6$, 28 °C): *δ* 3.34–4.64 (br, 66H), 7.17 (br, 6H), 7.53–7.73 (br, 22H), 8.16 (br, 2H), 8.46 (br, 2H), 11.64 (br, 3H). ^{13}C NMR (125 MHz, DMSO-*d*$_6$, 28 °C): *δ* 35.7, 47.2, 49.6, 51.0, 51.4, 62.5, 68.5, 68.8, 69.5, 69.7, 71.7, 108.1, 114.4, 125.5, 127.8, 128.3, 129.1, 129.9, 131.9, 132.1, 135.6, 137.4, 141.3, 151.7, 157.0, 160.9, 166.6, 194.3. MALDI-TOF-MS (*m/z*): calcd. for $C_{88}H_{112}N_{21}O_{17}S$ [M – 3HCl + H$^+$], 1734.85; found, 1735.69.

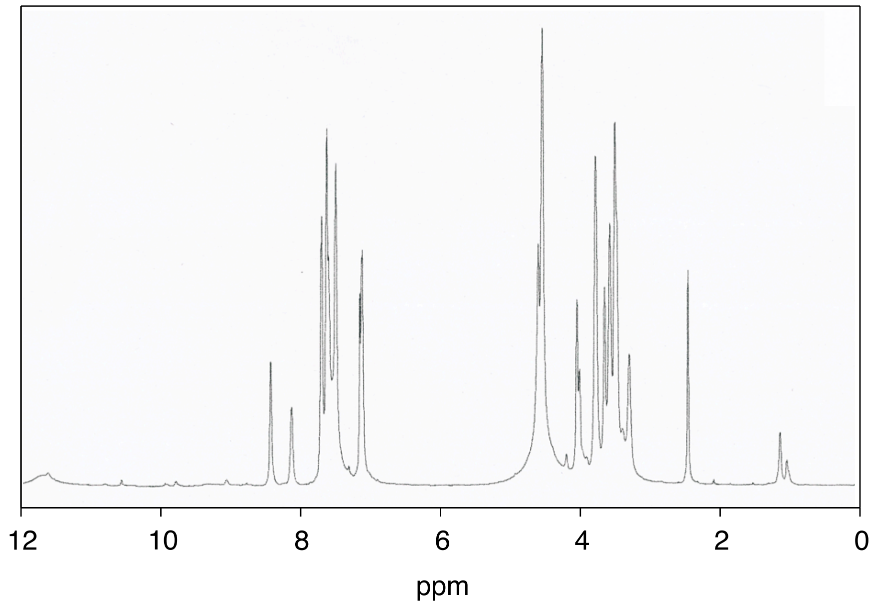

Supplementary Fig. 1. ^{1}H NMR spectrum (500 MHz) of Glue$^{CO_2-}$ in DMSO-d_6 at 28 °C.

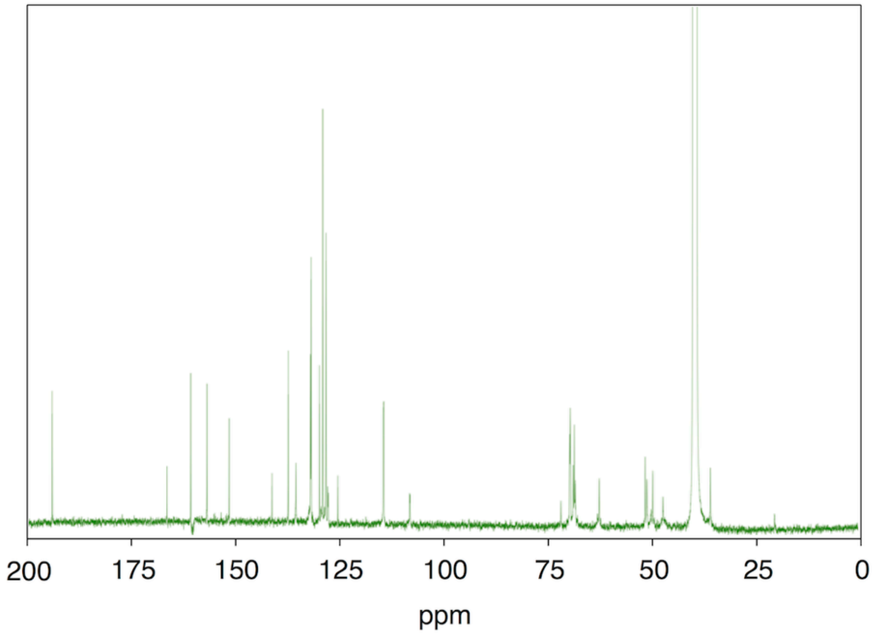

Supplementary Fig. 2. ^{13}C NMR spectrum (125 MHz) of Glue$^{CO_2-}$ in DMSO-d_6 at 28 °C.

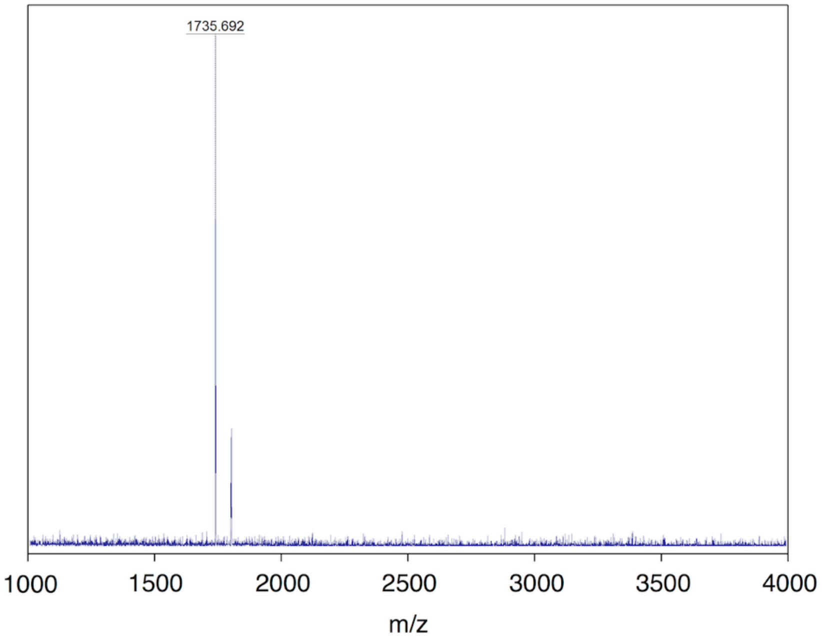

Supplementary Fig. 3. MALDI-TOF mass spectrum of Glue$^{CO_2-}$.

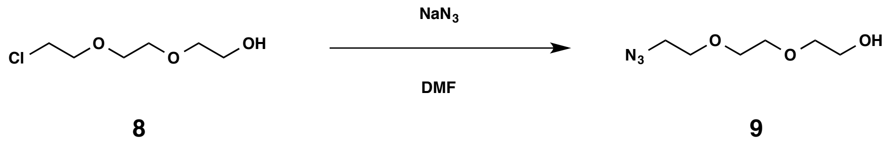

Compound **9**: To a dry DMF (20 ml) solution of 2-(2-(2-chloroethoxy)ethoxy)ethanol (**8**, 2.5 g, 14.8 mmol) was added sodium azide (NaN_3, 1.2 g, 17.8 mmol), and the mixture was stirred for 12 h at 65 °C. The reaction mixture was diluted with ether (50 ml) and filtered off from an insoluble fraction. The filtrate was washed with water (30 ml × 2) followed by brine (40 ml × 2). A separated organic extract was dried over Na_2SO_4 and filtered off from an insoluble fraction. The filtrate was evaporated to dryness under reduced pressure, and the residue was chromatographed on silica gel with AcOEt/hexane (1/1) as an eluent to allow for the isolation of **9** as a colorless oil (2.31 g, 89%).

^1H NMR (500 MHz; $CDCl_3$): δ 2.34 (br, 1H), 3.40 (t, J = 5.0 Hz, 2H), 3.62 (t, J = 5.0 Hz, 2H), 3.67–3.76 (m, 8H). ^{13}C NMR (125 MHz; $CDCl_3$): δ 50.7, 61.8, 70.1, 70.5, 70.7, 72.6.

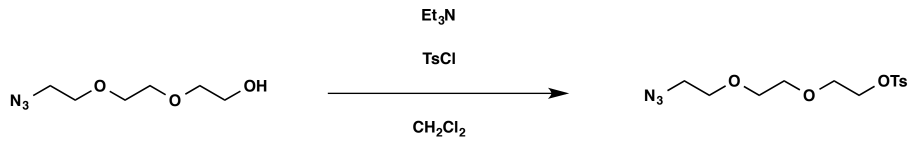

9 **10**

Compound **10**: To a dichloromethane (CH_2Cl_2, 50 ml) solution of a mixture of **9** (2.0 g, 11.4 mmol) and Et_3N (2.4 ml, 17.1 mmol) was dropwisely added p-toluenesulfonyl chloride (TsCl, 3.26 g, 17.1 mmol), and the mixture was stirred for 2 h at 0 °C and then for 2 h at room temperature. The reaction mixture was evaporated to dryness under reduced pressure, and the residue was diluted with CH_2Cl_2 (50 ml) and washed with hydrochloric acid (3%, 50 ml × 2), saturated aqueous $NaHCO_3$ (50 ml × 2) and then brine (50 ml × 2). An organic extract separated was dried over Na_2SO_4 and filtered off from an insoluble fraction. The filtrate was evaporated to dryness under reduced pressure, affording **10** as a pale yellow oil (3.7 g, quant.).

^{1}H NMR (500 MHz; $CDCl_3$): δ 2.45 (s, 3H), 3.67 (t, J = 5.0 Hz, 2H), 3.60 (s, 4H), 3.64 (t, 2H), 3.70 (t, 2H), 4.16 (t, J = 5.0 Hz, 2H), 7.35 (d, J = 8.0 Hz, 2H), 7.80 (d, J = 8.0 Hz, 2H). ^{13}C NMR (125 MHz; $CDCl_3$): δ 21.7, 50.7, 68.8, 69.4, 70.1, 70.7, 70.8, 128.0, 129.9, 133.0, 144.9.

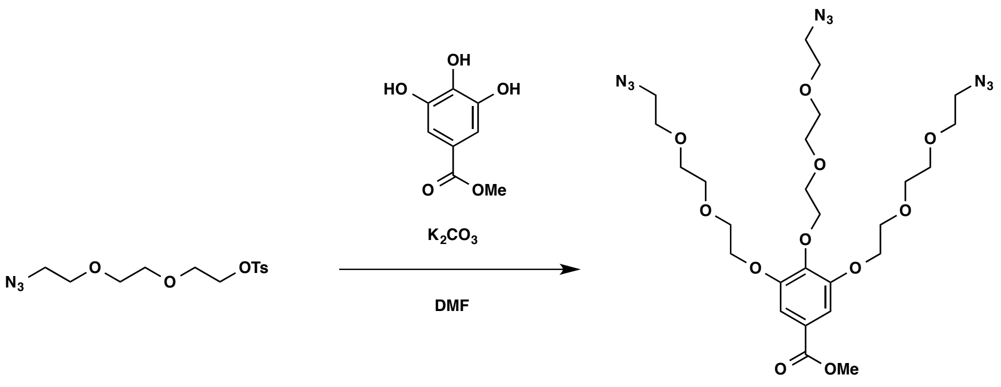

10 **11**

Compound **11**: To a DMF (20 ml) suspension of a mixture of methyl gallate (0.41 g, 2.25 mmol) and K_2CO_3 (1.55 g, 11.2 mmol) was added **10** (3.7 g, 11.2 mmol), and the mixture was stirred for 24 h at 80 °C. The reaction mixture was poured into water (100 ml) and extracted with CH_2Cl_2 (50 ml × 4). A separated organic extract was dried over Na_2SO_4 and filtered off from an insoluble fraction. The filtrate was evaporated to dryness under reduced pressure, and the residue was chromatographed on silica gel with AcOEt/hexane (1/3 to 2/1) as an eluent to allow for the isolation of **11** as a pale yellow oil (1.1 g, 75%).

^{1}H NMR (500 MHz; $CDCl_3$): δ 3.39 (m, 6H), 3.60–3.75 (m, 24H), 3.87 (s, 3H), 7.30 (s, 2H). ^{13}C NMR (125 MHz; $CDCl_3$): δ 50.8, 52.3, 68.9, 69.7, 70.1, 70.7, 70.8, 70.9, 72.5, 109.1, 125.0, 142.6, 152.4, 166.7.

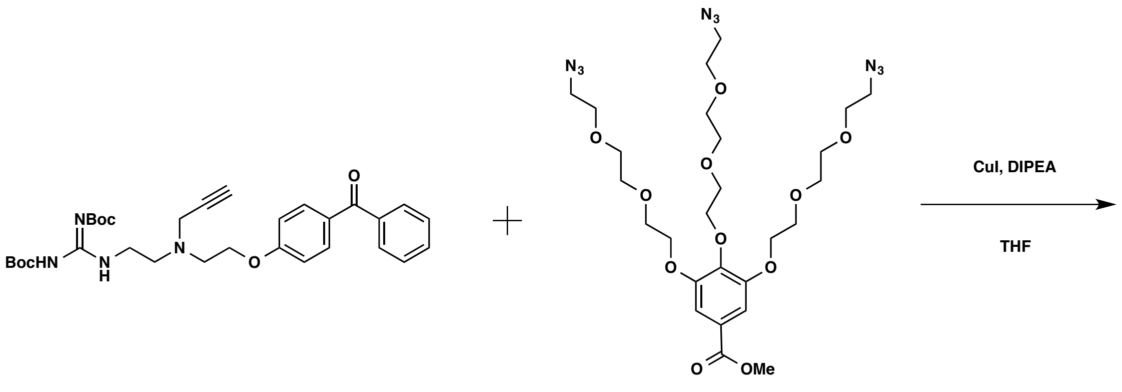

5 **11**

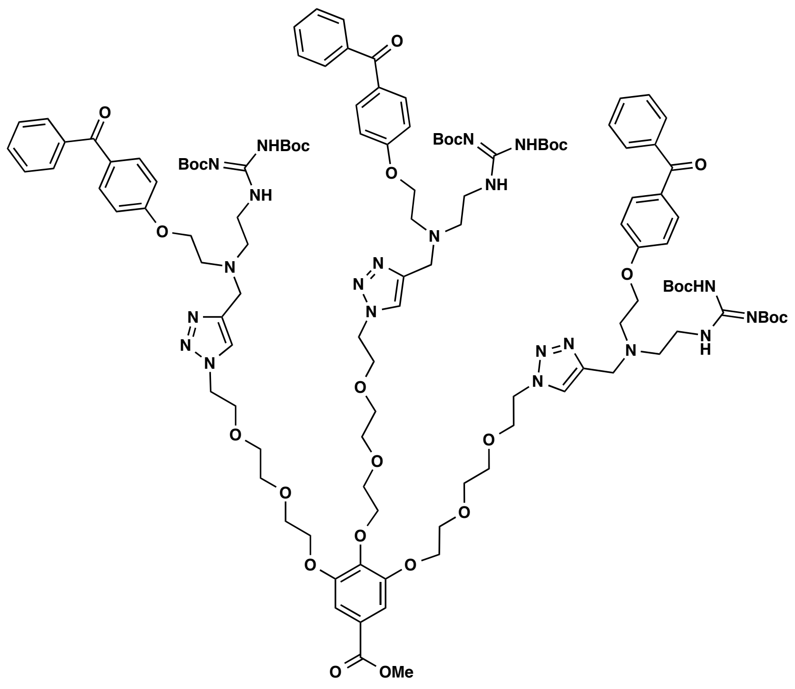

12

Compound **12**: To a THF (60 ml) solution of a mixture of **5** (470 mg, 832 μmol), **11** (136 mg, 208 μmol)[S7], and DIPEA (181 μl, 1.7 mmol) was added copper iodide (158 mg, 832 μmol), and the mixture was stirred for 17 h at room temperature. The reaction mixture was extracted with AcOEt, and washed with saturated aqueous NH_4Cl and brine. A separated organic extract was dried over Na_2SO_4 and filtered off from an insoluble fraction. The filtrate was subjected to recycling preparative GPC using $CHCl_3$ as an eluent to isolate **12** as a green solid at a 21% yield (100 mg).

^{1}H NMR (500 MHz, $CDCl_3$, 24 °C): δ 1.43–1.46 (s, 54H), 2.80–2.97 (br, 12H), 3.54–4.13 (br, 48H), 4.47 (br, 6H), 6.90–6.92 (br, 6H), 7.45–7.75 (br, 26H), 8.73 (s, 3H), 11.43 (br, 3H). ^{13}C NMR (125 MHz, $CDCl_3$, 24 °C): δ 28.1, 28.4, 29.8, 38.7, 49.3, 50.3, 50.8, 52.2, 52.5, 66.9, 68.8, 69.6, 69.9, 70.5, 72.5, 79.5, 83.1, 108.9, 114.1, 124.1, 125.4, 128.3, 129.8, 130.2, 132.1, 132.6, 138.1, 138.3, 152.3, 153.0, 156.1, 162.0, 162.5, 163.4, 195.6. MALDI-TOF-MS (*m/z*): calcd. for $C_{89}H_{114}N_{21}O_{17}S$ [M – 6Boc + H^+], 1748.87; found, 1749.83.

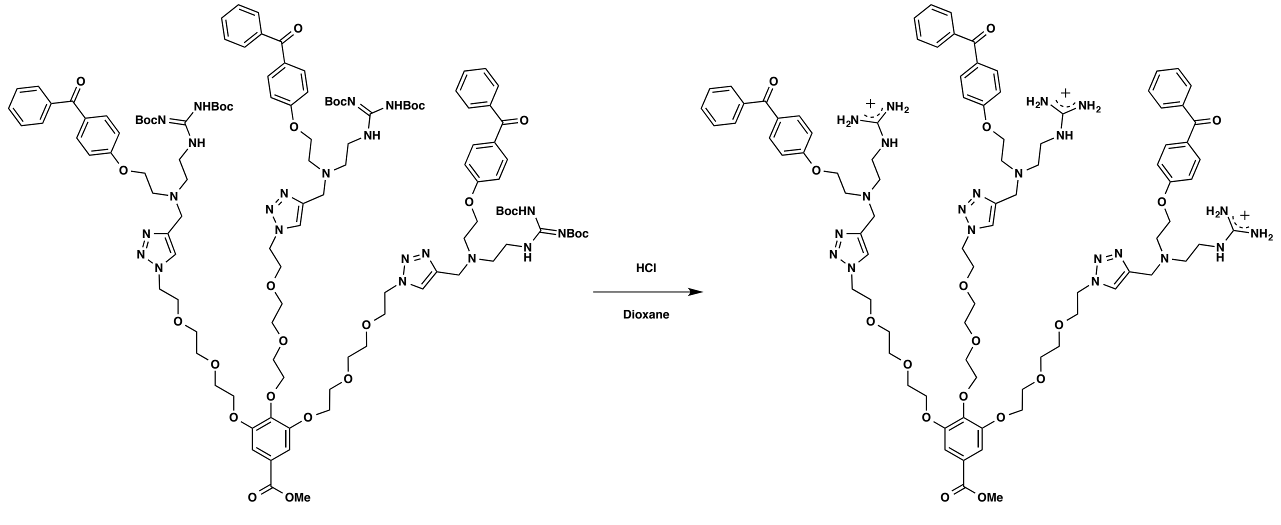

12 Glue^{CO_2Me}

Glue^{CO_2Me}: To a 1,4-dioxane/Et_2O (1/1) solution (10 ml) of HCl (2 M) was added **12** (6.5 mg 2.8 μmol), and the mixture was stirred for 4.5 h at room temperature. Then, the reaction mixture was evaporated to dryness under reduced pressure. The residue was dissolved in water and then reprecipitated in 1,4-dioxane to isolate Glue^{CO_2Me} as a yellow solid with an 83% yield (4.3 mg).

^{1}H NMR (500 MHz, D_2O, 27 °C): δ 3.54–3.97 (br, 57H), 4.31 (br, 6H), 4.66 (br, 6H), 6.88–6.90 (br, 8H), 7.49–7.62 (br, 22H), 8.29 (br, 2H). ^{13}C NMR (125 MHz, DMSO-d_6, 27 °C): δ 48.0, 49.3, 49.7, 51.3, 52.3, 66.4, 68.4, 68.7, 69.5, 69.7, 71.9, 108.2, 114.3, 124.1, 124.4, 128.3, 128.3, 128.4, 129.2, 129.3, 132.1, 137.7, 141.7, 142.7, 151.9, 157.1, 162.0, 165.7, 194.3. MALDI-TOF-MS (*m/z*): calcd. for $C_{89}H_{114}N_{21}O_{17}S$ [M – 3HCl + H^+], 1748.87; found, 1749.22.

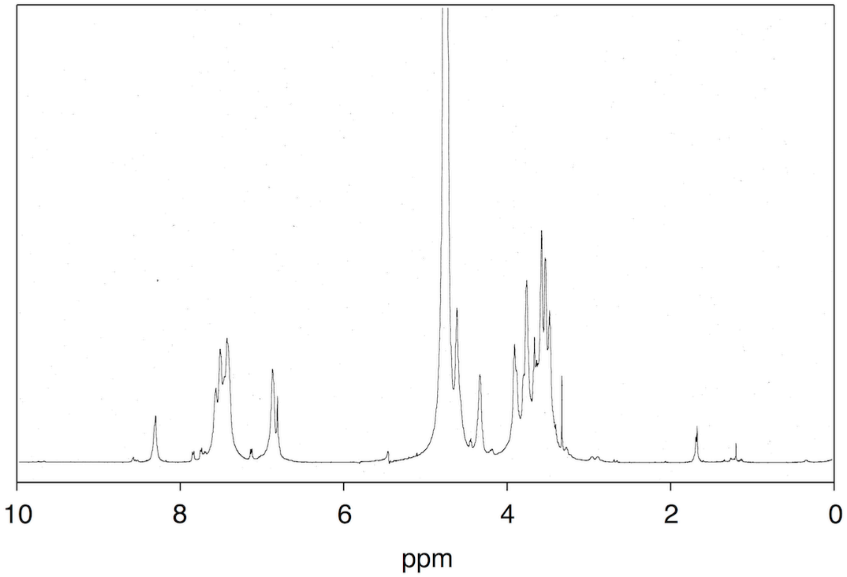

Supplementary Fig. 4. ^{1}H NMR spectrum (500 MHz) of Glue^{CO_2Me} in D_2O at 27 °C.

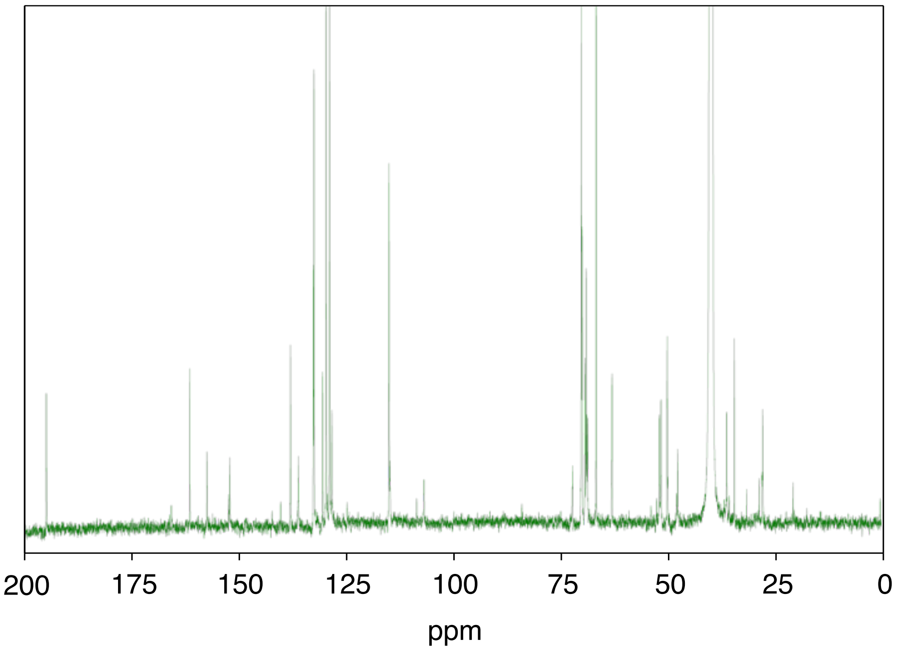

Supplementary Fig. 5. ^{13}C NMR spectrum (125 MHz) of Glue^{CO_2Me} in DMSO-d_6 at 27 °C.

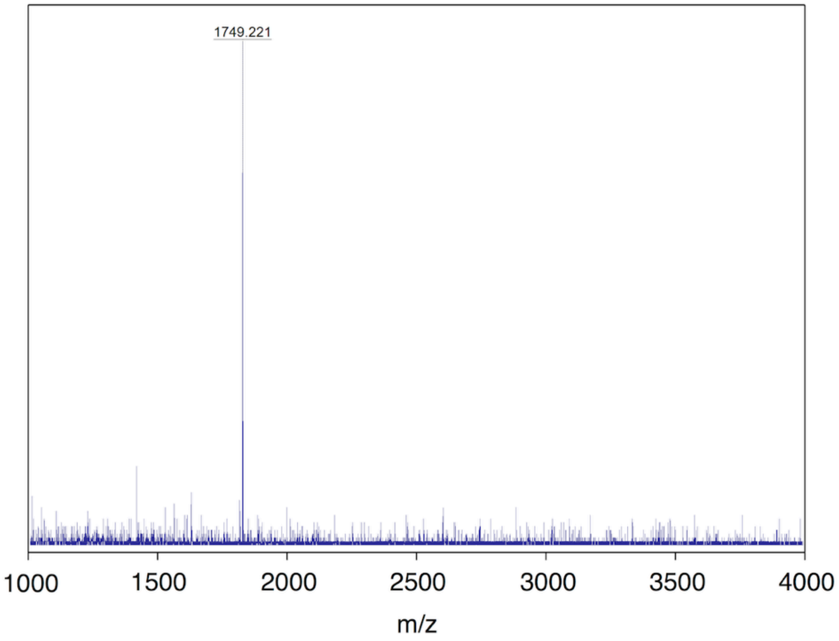

Supplementary Fig. 6. MALDI-TOF mass spectrum of Glue^{CO_2Me}.

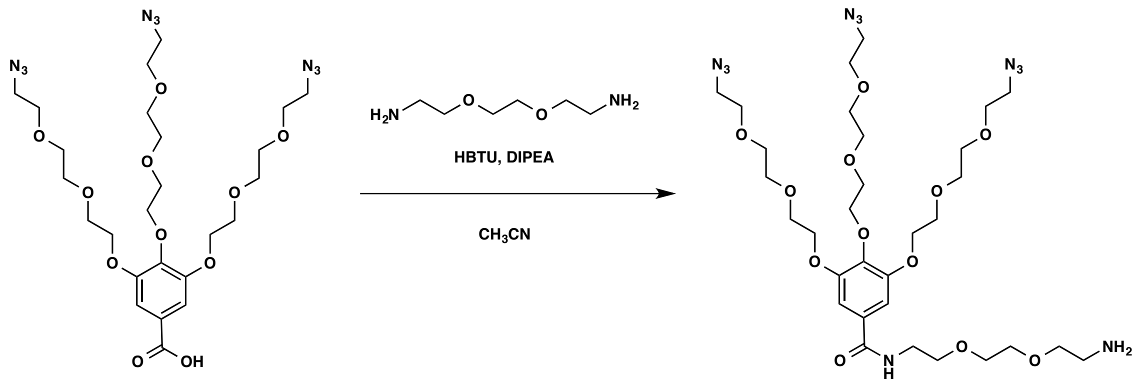

Compound **13.** To an acetonitrile (10 ml) solution of **6** (1.00 g, 1.56 mmol) was added 1-[bis(dimethylamino)methylene]-1H-benzotriazolium 3-oxide hexafluorophosphate (HBTU, 383 mg 1.64 mmol), and the mixture was stirred for 5 min at room temperature. Then, the resulting mixture was added to an acetonitrile (15 ml) solution of a mixture of 2,2'-(ethylenedioxy)bis(ethylamine) (2.29 ml, 15.6 mmol) and DIPEA (172 μl, 1.56 mmol), and the resultant mixture was stirred for 1 h at room temperature. The reaction mixture was evaporated to dryness under reduced pressure, and the residue was extracted with $CHCl_3$ (30 ml) and washed with water (30 ml × 3). The organic extract separated was dried over Na_2SO_4 and evaporated to dryness under reduced pressure to give **13** as yellow oil in 40% yield (474 mg).

^{1}H NMR (500 MHz, $CDCl_3$): δ 2.82−2.85 (m, 2H), 3.34−3.38 (t, J = 5.1 Hz, 6H), 3.48−3.86 (m, 34H), 4.16−4.21 (m, 6H), 7.07 (s, 2H). ^{13}C NMR (125 MHz, $CDCl_3$): δ 39.9, 50.7, 69.8, 70.0, 70.1, 72.5, 107.3, 129.8, 152.3, 167.0. MALDI-TOF-MS (*m/z*): calcd. for $C_{31}H_{53}N_{11}O_{12}Na$ [M + Na^+], 794.38; found, 794.41.

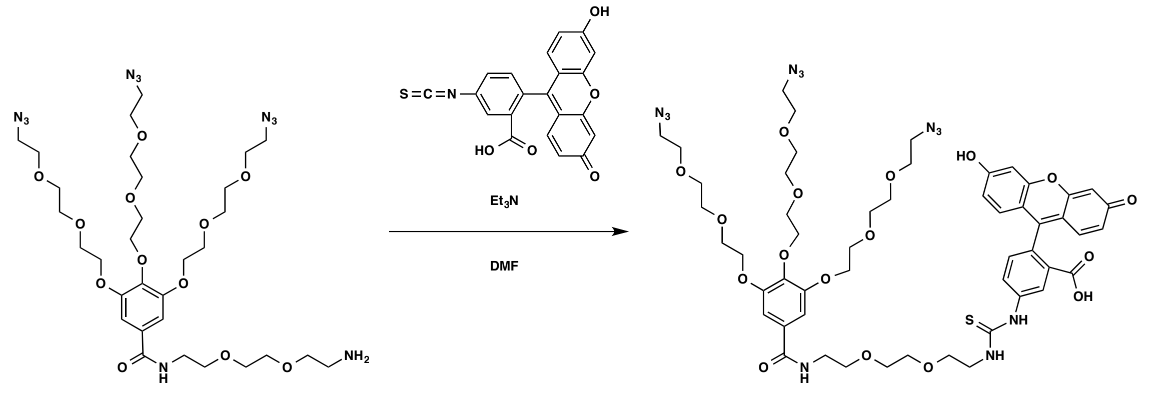

Compound **14.** To a DMF (3 ml) solution of a mixture of **13** (216 mg, 280 μmol) and Et_3N (39.0 μl, 280 μmol) was added fluorescein isothiocyanate-isomer I (218 mg, 560 μmol), and the mixture was stirred overnight at room temperature. Then, the reaction mixture was extracted with $CHCl_3$/methanol (30 ml, 9/1) and washed with water (30 ml) followed by brine (30 ml). An organic extract separated was dried over Na_2SO_4 and filtered off from an insoluble fraction. The filtrate was evaporated to dryness under reduced pressure, and the residue was dissolved in methanol and reprecipitated with water to allow isolation of **14** as yellow solid in 68% yield (220 mg).

^{1}H NMR (500 MHz, $(CD_3)_2CO$): δ 3.23 (m, 6H), 3.35−3.85 (m, 36H), 4.02 (m, 6H), 6.44−7.75 (m, 11H), 7.96 (br, 1H), 8.30 (br, 1H), 9.67 (br, 1H). ^{13}C NMR (125 MHz, $(CD_3)_2CO$): δ 40.7, 45.0, 51.2, 69.5, 70.1, 70.4, 70.7, 71.0, 71.1, 71.2, 73.0, 84.3, 103.2, 107.3, 111.4, 113.2, 118.0, 124.6, 128.0, 129.8, 129.9, 130.3, 141.7, 142.2, 148.5, 153.0, 153.1, 160.0, 168.0, 169.2, 182.0. MALDI-TOF-MS (*m/z*): calcd. for $C_{52}H_{63}N_{12}O_{17}K$ [M + K^+], 1199.39; found, 1200.27.

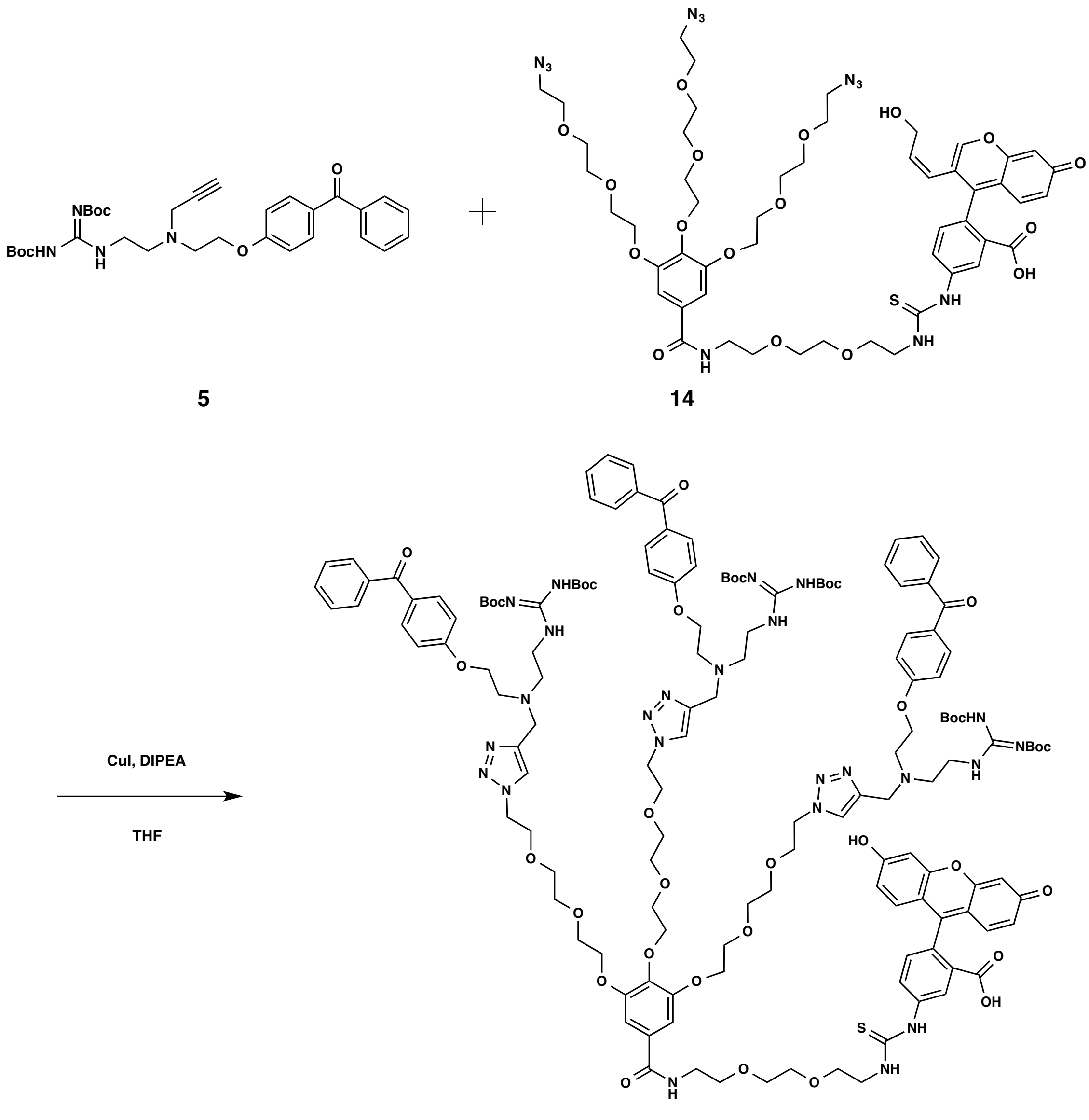

15

Compound **15**: To a THF (50 ml) solution of a mixture of **5** (215 mg, 380 μmol), **14** (110 mg, 95 μmol), and DIPEA (84 μl, 760 μmol) was added copper iodide (72 mg, 380 μmol), and the mixture was stirred overnight at room temperature. The reaction mixture was extracted with AcOEt, and washed with saturated aqueous NH_4Cl and brine. A separated organic extract was dried over Na_2SO_4 and filtered off from an insoluble fraction. The filtrate was subjected to recycling preparative GPC using $CHCl_3$ as an eluent to isolate **15** as a yellow solid with a 56% yield (151 mg).

^{1}H NMR (500 MHz, DMSO-d_6, 23 °C): δ 1.35–1.39 (s, 54H), 2.67–2.84 (br, 12H), 3.50–4.13 (br, 60H), 4.46 (br, 6H), 6.58–6.67 (br, 5H), 7.01-7.17 (br, 9H), 7.52-7.64 (br, 23H), 7.97 (br, 4H), 8.48 (s, 3H), 10.13 (br, 1H), 11.43 (br, 3H). ^{13}C NMR (125 MHz, $CDCl_3$, 27 °C): δ 28.1, 28.3, 29.7, 31.3, 38.7, 49.5, 50.4, 52.2, 52.5, 67.0, 69.3, 69.5, 70.6, 79.3, 82.9, 107.1, 114.1, 115.6, 124.0, 124.9, 128.3, 129.8, 130.1, 132.0, 132.6, 138.3, 140.6, 144.9, 152.2, 153.0, 156.0, 158.6, 162.1, 162.5, 163.5, 195.6. MALDI-TOF-MS (*m/z*): calcd. for $C_{115}H_{137}N_{24}O_{23}S$ [M – 6Boc + H^+], 2254.00; found, 2254.57.

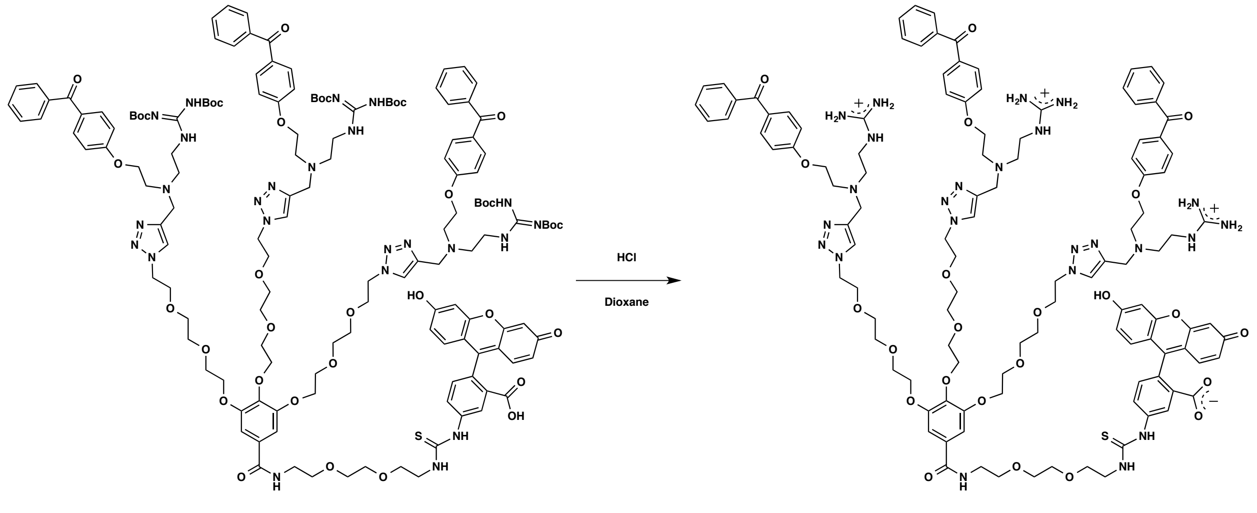

Glue^{FITC}: To a 1,4-dioxane (10 ml) solution of HCl (4 M) was added **15** (82 mg 29 μmol), and the mixture was stirred for 3 h at room temperature. Then, the reaction mixture was evaporated to dryness under reduced pressure. The residue was dissolved in water and then reprecipitated in 1,4-dioxane to isolate Glue^{FITC} as yellow a solid at a 61% yield (44 mg).

^{1}H NMR (500 MHz, DMSO-d_6, 24 °C): δ 2.72–2.88 (br, 12H), 3.54–4.64 (br, 66H), 6.59–6.75 (br, 4H), 7.15–7.21 (br, 7H), 7.35 (br, 2H), 7.53–7.73 (br, 23H), 8.12 (br, 3H), 8.45 (br, 2H), 11.64 (br, 3H). ^{13}C NMR (125 MHz, DMSO-d_6, 24 °C): δ 35.8, 47.2, 49.7, 51.1, 51.5, 60.1, 62.6, 68.3, 68.6, 68.9, 69.6, 69.7, 71.8, 102.2, 106.3, 112.8, 114.6, 127.9, 128.3, 128.4, 129.2, 130.0, 132.0, 132.2, 135.6, 137.5, 139.7, 151.6, 157.0, 161.0, 162.3, 165.5, 194.4. MALDI-TOF-MS (*m/z*): calcd. for $C_{115}H_{136}N_{24}O_{22}S$ [M – 3HCl – OH + H$^+$], 2238.01; found, 2238.04, calcd. for $C_{115}H_{137}N_{24}O_{23}S$ [M – 3HCl + H$^+$], 2254.00; found, 2253.30.

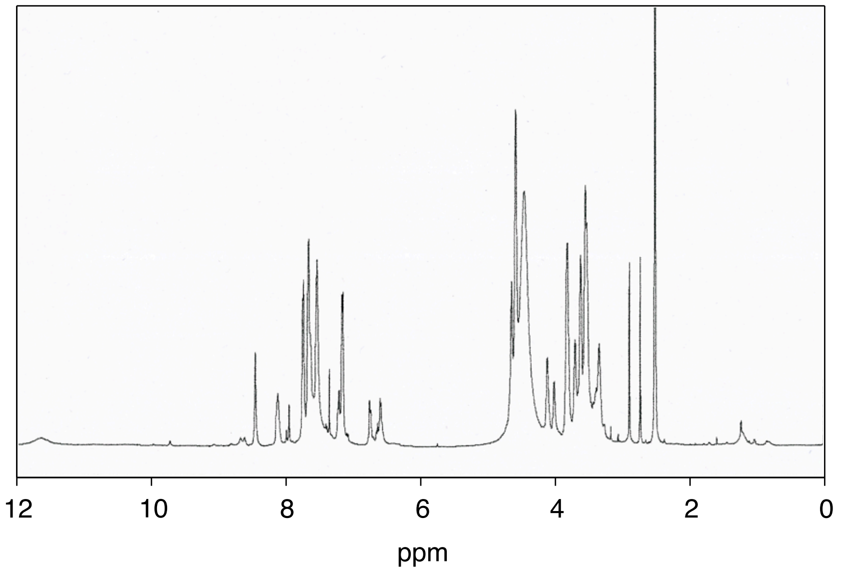

Supplementary Fig. 7. ^{1}H NMR spectrum (500 MHz) of Glue^{FITC} in DMSO-d_6 at 24 °C.

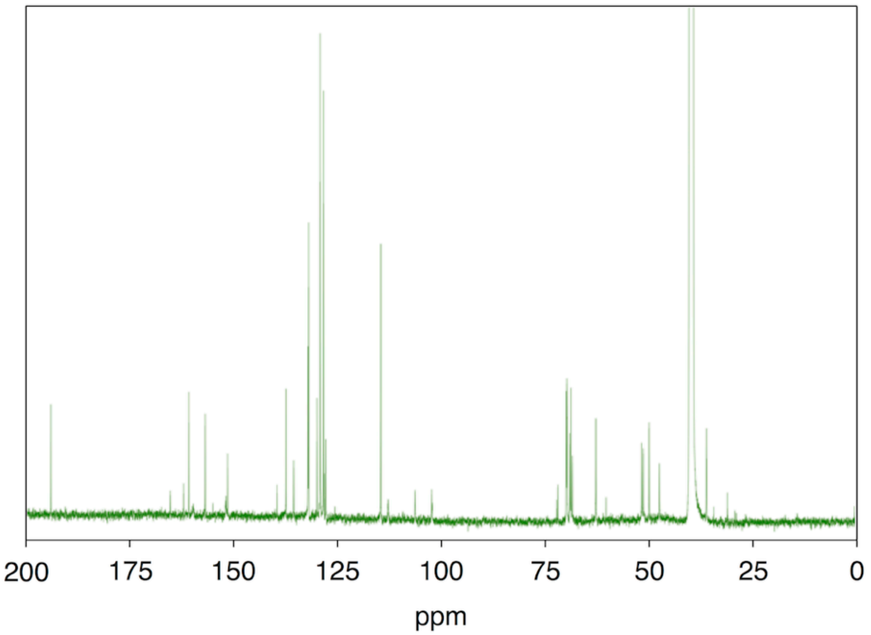

Supplementary Fig. 8. ^{13}C NMR spectrum (125 MHz) of Glue^{FITC} in DMSO-d_6 at 24 °C.

1-4. MD simulation

Folding of Glue$^{CO_2-}$ in aqueous solution:

An atomistic molecular model of Glue$^{CO_2-}$ was created and parameterized according to the general AMBER force field (GAFF)[S8]. As a first step, one Glue$^{CO_2-}$ molecule was placed in the center of a periodic simulation box filled with explicit TIP3P water molecules[S9]. Neutralizing chloride (3) and sodium (1) counter ions were added to neutralize the system and to reach 0.15 M of ionic strength. After preliminary minimization, the system was initially heated to 37 °C via 1 ns of MD simulation conducted in NVT (constant N: number of atoms, V: volume, T: temperature) conditions. Then, Glue$^{CO_2-}$ was equilibrated for 200 ns of MD simulations under constant NPT (N: number of atoms, P: pressure, T: temperature) periodic boundary conditions at 37 °C and 1 atm using a 2 fs time step and a 1.2 nm cut-off.

Arrangements of Glue$^{CO_2-}$ and THD$_{GTP*}$:

The [THD$_{GTP*}$]$_{3*}$ system was obtained from a full MT model built using the Protein Data Bank (PDB) structure for THD$_{GTP*}$ (PDB code: 3J6E [https://www.rcsb.org/structure/3j6e])[S10]. Missing residues in the THD$_{GTP*}$ structure were compensated from another THD structure (PDB code: 1TUB [https://www.rcsb.org/structure/1TUB])[S11]. In the [THD$_{GTP*}$]$_{3*}$ system, 30 equivalents of pre-equilibrated (folded) Glue$^{CO_2-}$ molecules were placed randomly in a periodic simulation boxes. As a control, the [THD$_{GTP*}$]$_{3*}$ system without Glue$^{CO_2-}$ was also simulated. All MD simulations have been conducted in explicit TIP3P water molecules[S9] and in the presence of the necessary number of counterions to neutralize the systems.

Adhesions of Glue$^{CO_2^-}$ on THD$_{GTP*}$:

After preliminary minimization, all systems were first heated during two short MD runs under NVT (1 ns) and NPT (1 ns) periodic boundary conditions, during which the atoms of the THD$_{GTP*}$ were restrained (position restraint of 1000 kJ/mol/nm^2). During these phases, the system reached the simulation temperature of 37 °C, and the solvent density inside the simulation box was preadjusted. After this preliminary equilibration, each system underwent a short MD simulation (20 ns) under NVT conditions at 37 °C while the THD$_{GTP*}$ was maintained at a fixed position to allow Glue$^{CO_2^-}$ to approach the THD$_{GTP*}$ surface. Then, all restraints were removed, and all systems underwent 200 ns of MD simulation under NPT periodic boundary conditions at 37 °C and 1 atm. The AMBER99-ILDN force field was used to treat the protein topology[S12]. A cut-off of 1.2 for both electrostatic and van der Waals interactions was used in the MD simulations. The Particle-Mesh Ewald method was applied to treat with a 1.2 nm cut-off for the Lennard-Jones interactions[S13]. A *v-rescale* thermostat[S14] with a coupling time step of 0.1 ps and Parrinello-Rahman barostat[S15] with a reference pressure of 1 atm and coupling time step of 5.0 ps were used during the MD runs. The effects of Glue$^{CO_2^-}$ on the hydrophobicity and total solvent-accessible surface area of the THD$_{GTP*}$ surface were calculated with the GROMACS *gmx sasa* tool[S16]. Electrostatic potentials of the THD$_{GTP*}$ surface depending on the binding Glue$^{CO_2^-}$ were studied using the Adaptive Poisson-Boltzmann Solver (APBS) software package[S17]. All data have been extraced from the equilibrated phase MD trajectories.

Interactions of Glue$^{CO_2^-}$:

To quantify the strength of the interactions of Glue$^{CO_2^-}$, the radial distribution functions g(r) between key groups in the glues and in the tubulins were extracted from the MD trajectories. High and sharp peaks at short distances in g(r) identify a high relative probability to find groups close to

each other during the MD. This identifies the presence of strong and persistent interactions between groups, while no evident peaks and g(r) values <1 typically indicate no, or negligible, interactions (see Fig. 3j in the main manuscript). To assess the glue-tubulin interactions, g(r) curves were calculated between (i) the Gu^+ groups of $Glue^{CO_2^-}$ and the anionic amino acids (aspartic acid and glutamic acid) of THD_{GTP*}, (ii) the Gu^+ groups of $Glue^{CO_2^-}$ and the OH groups of neutral amino acids (serine, threonine, and tyrosine) of THD_{GTP*}, and (iii) the CO_2^- groups of $Glue^{CO_2^-}$ and the cationic amino acids (lysine and arginine) of THD_{GTP*}. In addition, the g(r) were calculated to investigate the nature of $Glue^{CO_2^-}$-to-$Glue^{CO_2^-}$ interactions. To this end, we estimated the g(r) for CO_2^- *vs.* Gu^+ groups belonging to different $Glue^{CO_2^-}$ molecules in the simulated systems.

Complete modeling data, structures and parameters used for, and extracted from simulations are available at https://zenodo.org/record/7070651#.Yx80t9JBxkg.

2. Supplementary Notes

2-1. Characterization of $NS_{GTP/GTP*}$

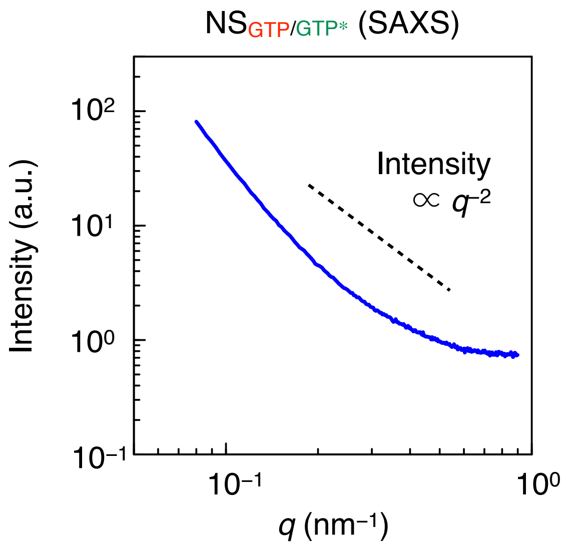

Supplementary Fig. 9. SAXS profile of $NS_{GTP/GTP*}$ (0.3 mg ml^{-1}) in PIPES buffer (100 mM PIPES, 1 mM $MgCl_2$, 250 μM GTP*, and 50 μM GTP, pH 6.8). The scattering intensity was proportional to q^{-2} in a small-q region[S18].

2-2. Self-assembly of THD in PIPES buffer containing a high mol% of GTP*

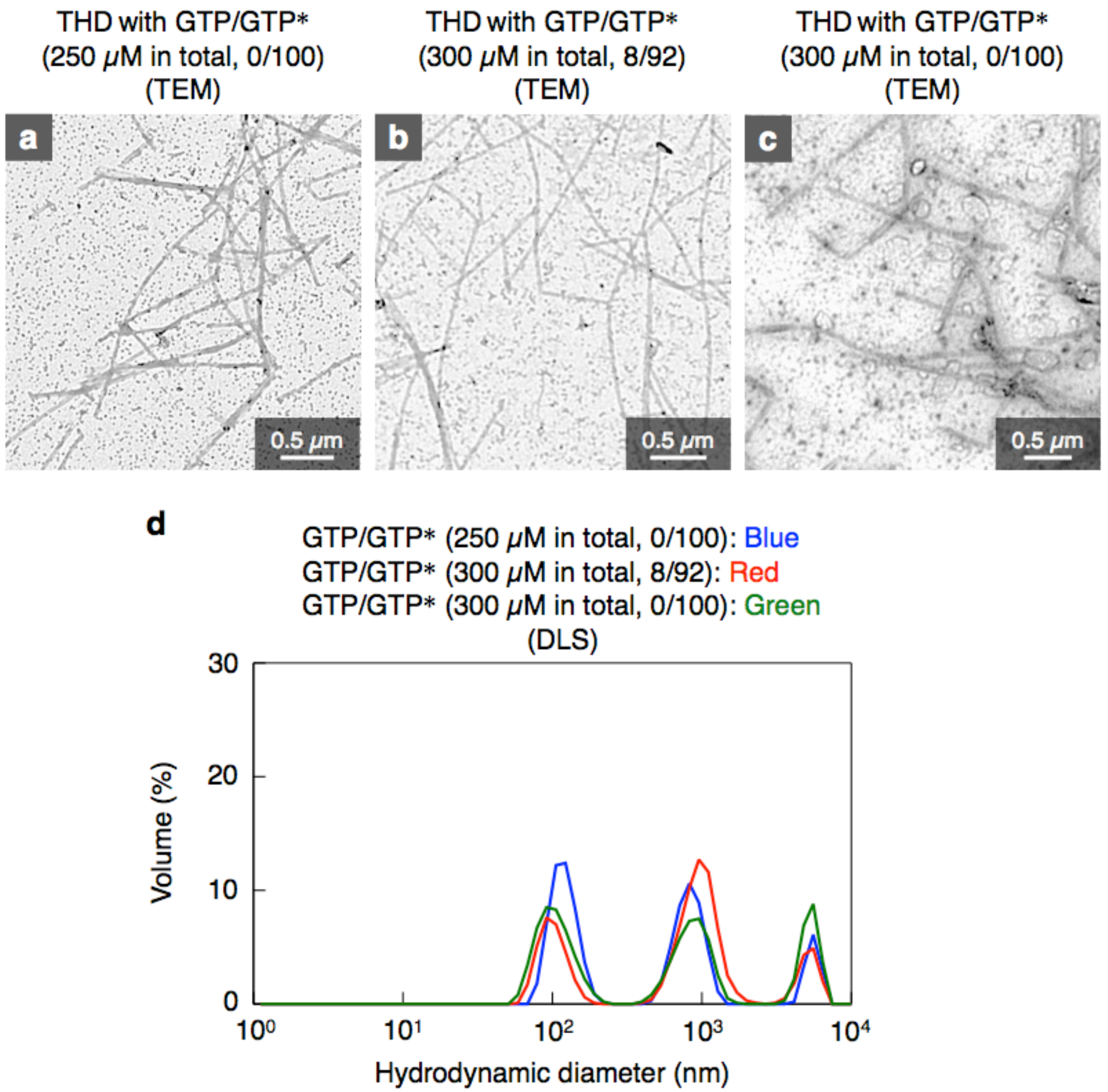

Supplementary Fig. 10. **a**–**c**, TEM images of THD_{GDP} (0.3 mg ml^{-1}) after an incubation with GTP/GTP* (0/250 μM; **a**), GTP/GTP* (25/275 μM; **b**), and GTP/GTP* (0/300 μM; **c**) at 37 °C for 30 min in PIPES buffer (100 mM PIPES and 1 mM $MgCl_2$, pH 6.8). **d**, DLS profiles of the THD samples incubated with GTP/GTP* (0/250 μM; blue), GTP/GTP* (25/275 μM; red), and GTP/GTP* (0/300 μM; green).

2-3. Self-assembly of THD in PIPES buffer containing a low mol% of GTP*

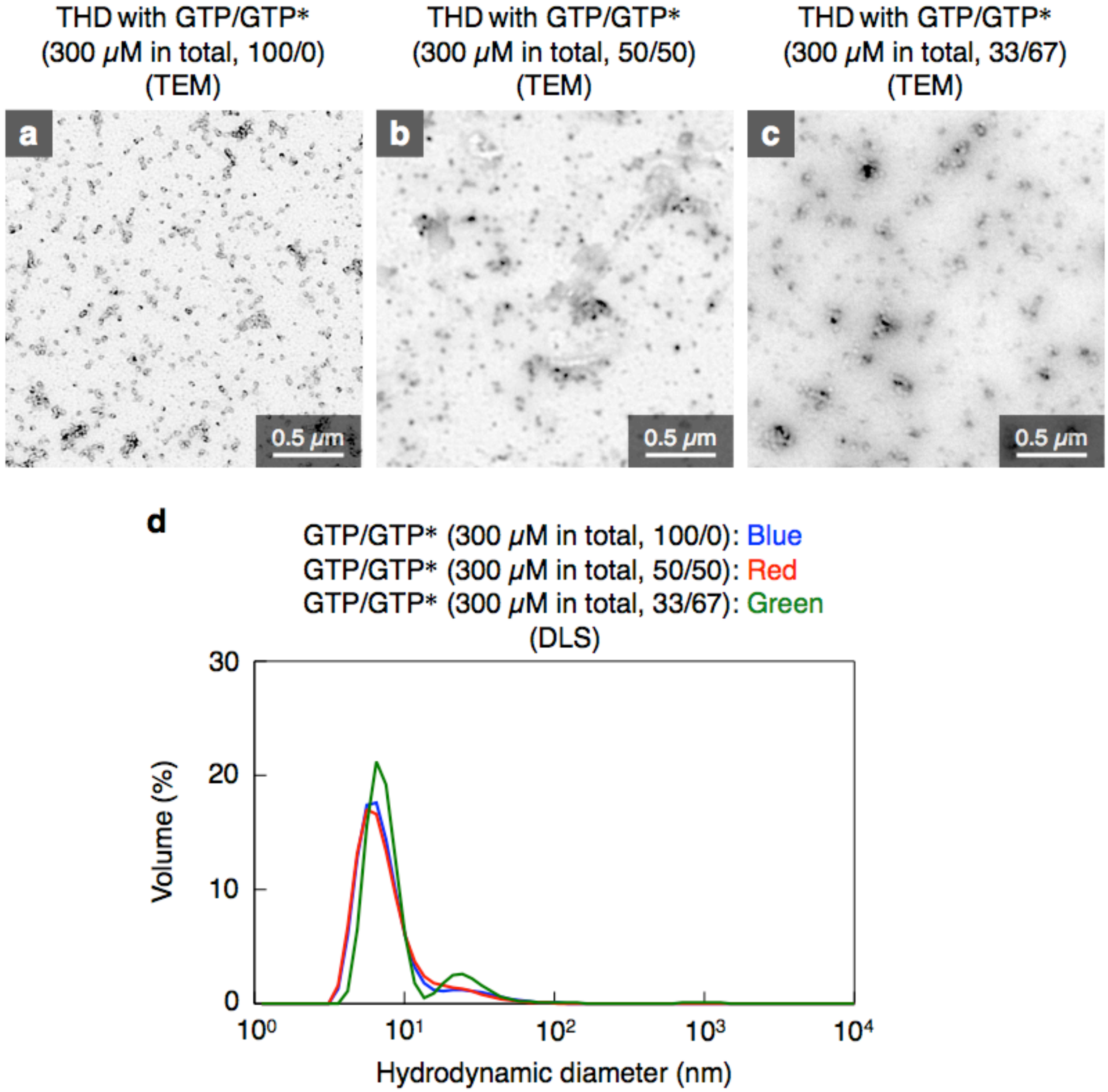

Supplementary Fig. 11. **a**–**c**, TEM images of THD (0.3 mg ml^{-1}) after an incubation with GTP/GTP* (300/0 μM; **a**), GTP/GTP* (150/150 μM; **b**), or GTP/GTP* (100/200 μM; **c**) at 37 °C for 30 min in PIPES buffer (100 mM PIPES and 1 mM $MgCl_2$, pH 6.8). **d,** DLS profiles of the THD samples incubated with GTP/GTP* (300/0 μM; blue), GTP/GTP* (150/150 μM; red), and GTP/GTP* (100/200 μM; green).

2-4. NMR measurement of $NS_{GTP/GTP*}$

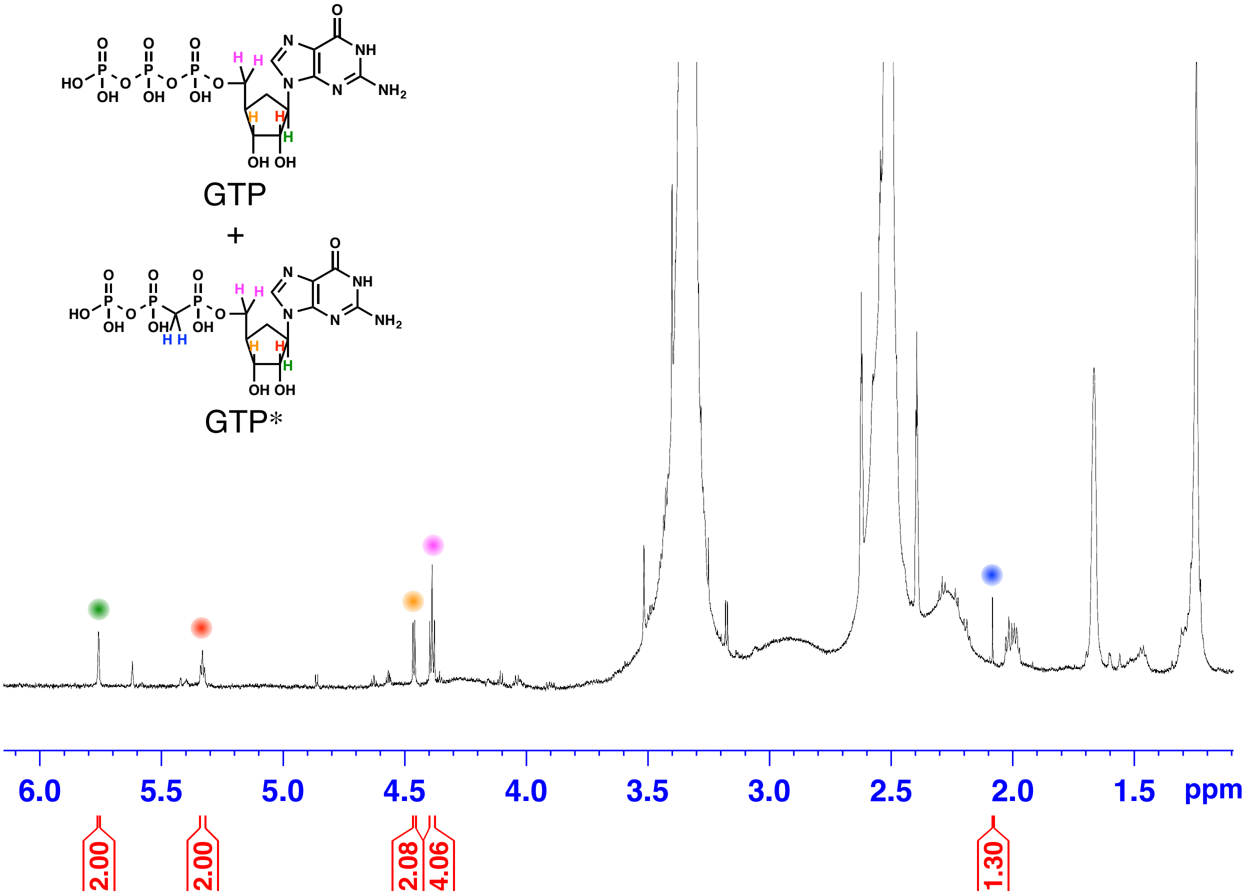

Supplementary Fig. 12. ^{1}H NMR spectrum (500 MHz) of $NS_{GTP/GTP*}$ in DMSO-d_6 at 25 °C. Since two GTP molecules are hybridized to THD_{GTP}, and one GTP and one GTP* molecule are hybridized to THD_{GTP*}, the NMR results show that 65% of THD_{GTP*} is contained in the $NS_{GTP/GTP*}$. Signals marked with blue, magenta, orange, red, and green circles are assignable to protons in GTP and GTP*, which are highlighted in the corresponding colors.

2-5. Self-assembly of THD in PIPES buffer containing a mixture of GTPγS and GTP*

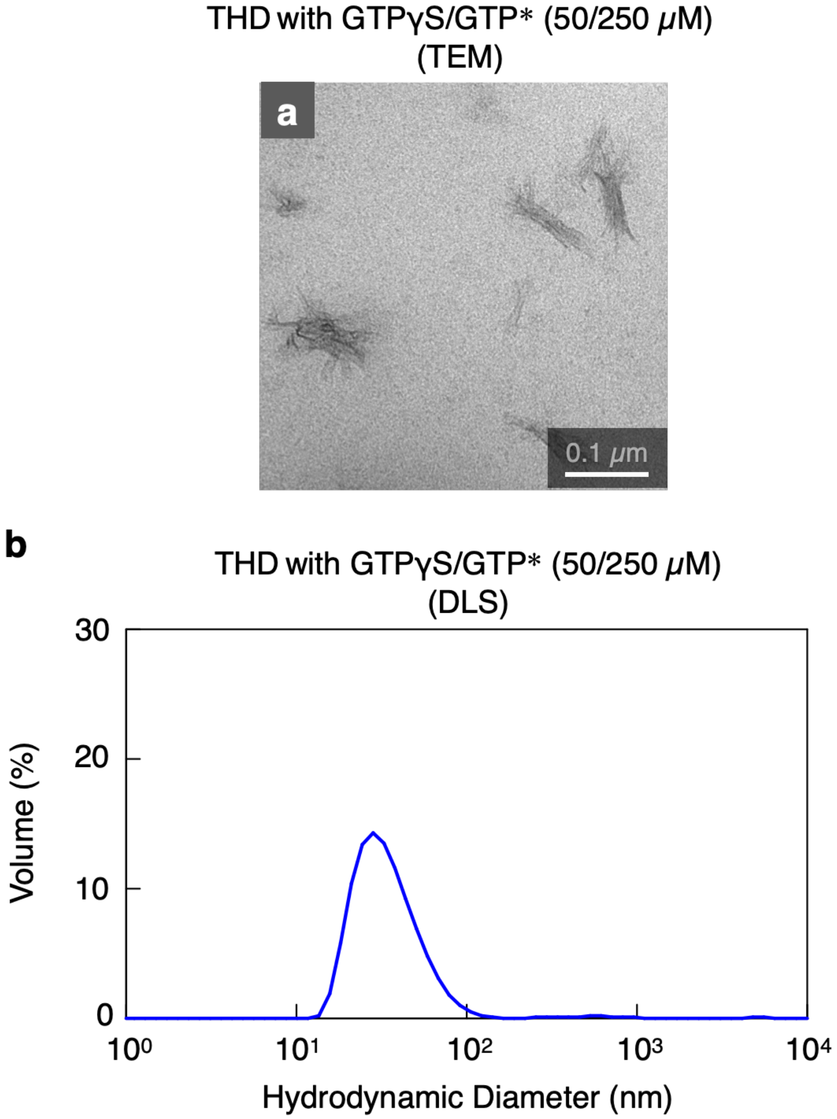

Supplementary Fig. 13. **a**, **b**, TEM image (**a**) and DLS profile (**b**) of THD (0.3 mg ml^{-1}) after an incubation with GTPγS/GTP* (50/250 μM) at 37 °C for 30 min in PIPES buffer (100 mM PIPES and 1 mM $MgCl_2$, pH 6.8).

2-6. Self-assembly of THD in PIPES buffer containing a mixture of GTP and GTPγS

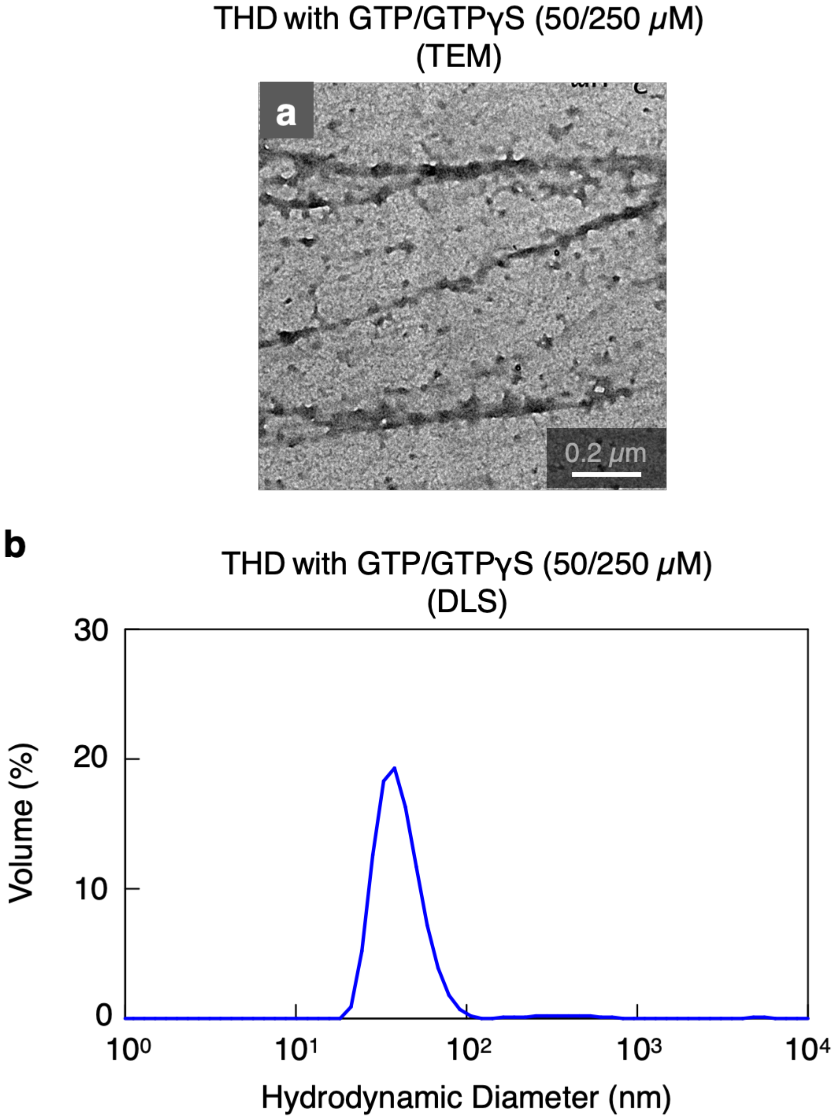

Supplementary Fig. 14. a, **b**, TEM image (**a**) and DLS profile (**b**) of THD (0.3 mg ml^{-1}) after an incubation with GTP/GTPγS (50/250 μM) at 37 °C for 30 min in PIPES buffer (100 mM PIPES and 1 mM $MgCl_2$, pH 6.8).

2-7. MT_{GTP*} treated with $Glue^{CO_2^-}$

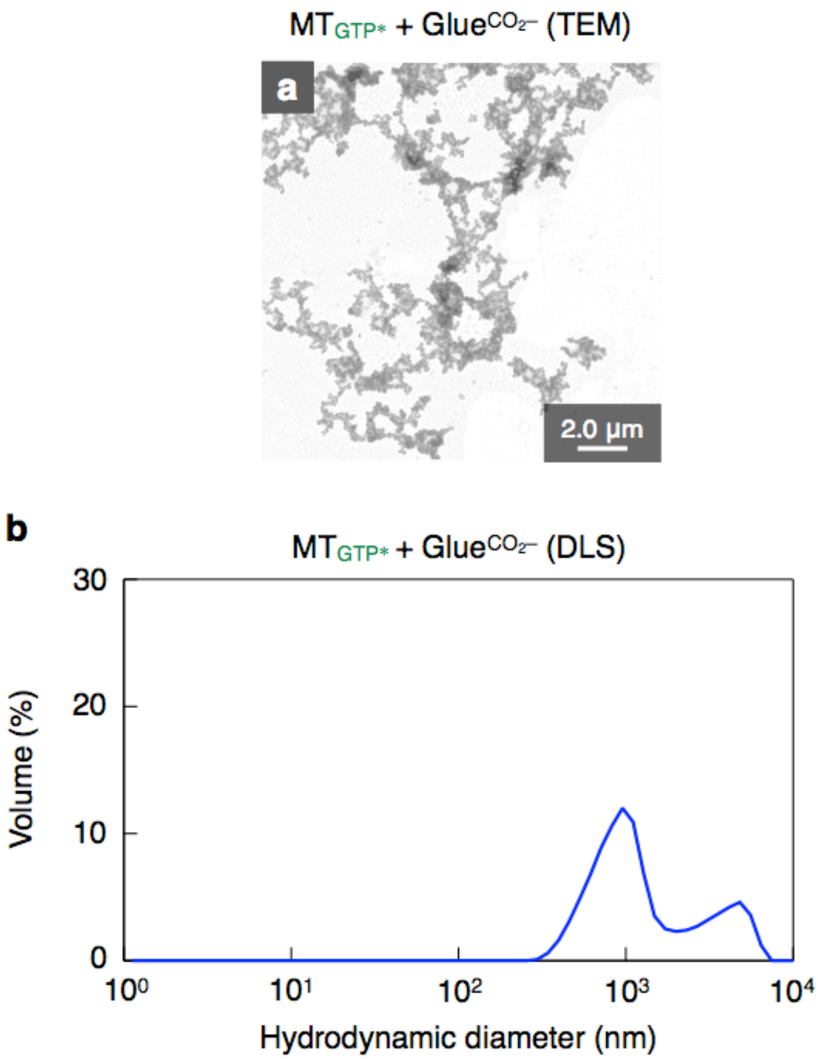

Supplementary Fig. 15. **a**, **b**, TEM image (**a**) and DLS profile (**b**) of MT_{GTP*} (13 μg ml^{-1}) in PIPES buffer (14 mM PIPES, 1 mM $MgCl_2$, and 0.2 mM GTP*, pH 6.8) after an incubation with $Glue^{CO_2^-}$ (100 μM) at 37 °C for 30 min.

2-8. THD_{GDP} treated with $Glue^{CO_2-}$

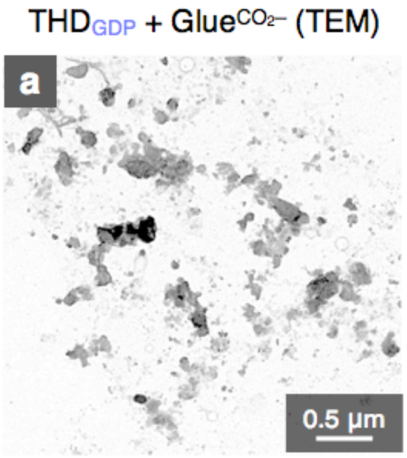

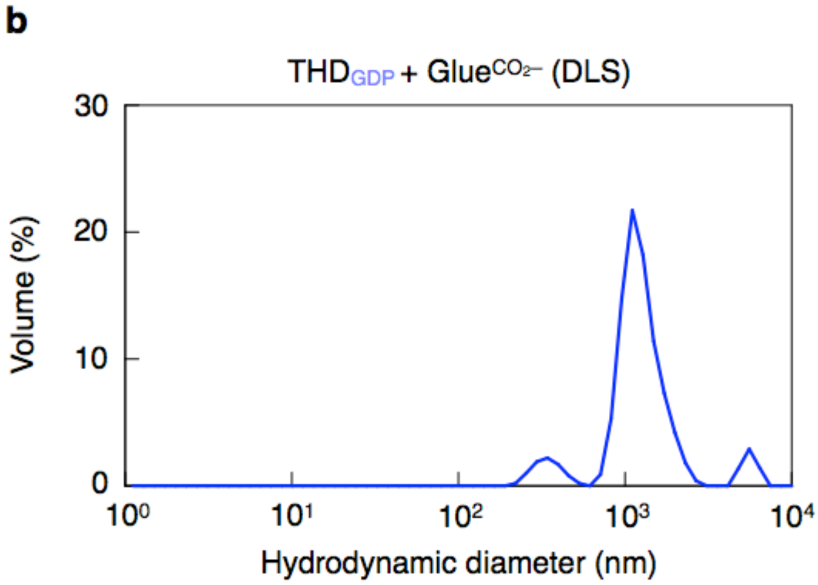

Supplementary Fig. 16. **a**, **b**, TEM image (**a**) and DLS profile (**b**) of THD_{GDP} (0.3 mg ml^{-1}) in PIPES buffer (100 mM PIPES and 1 mM $MgCl_2$, pH 6.8) after an incubation with $Glue^{CO_2-}$ (100 µM) at 37 °C for 30 min.

2-9. Photo-induced covalent fixation of Glue^{FITC} onto THD$_{GDP}$

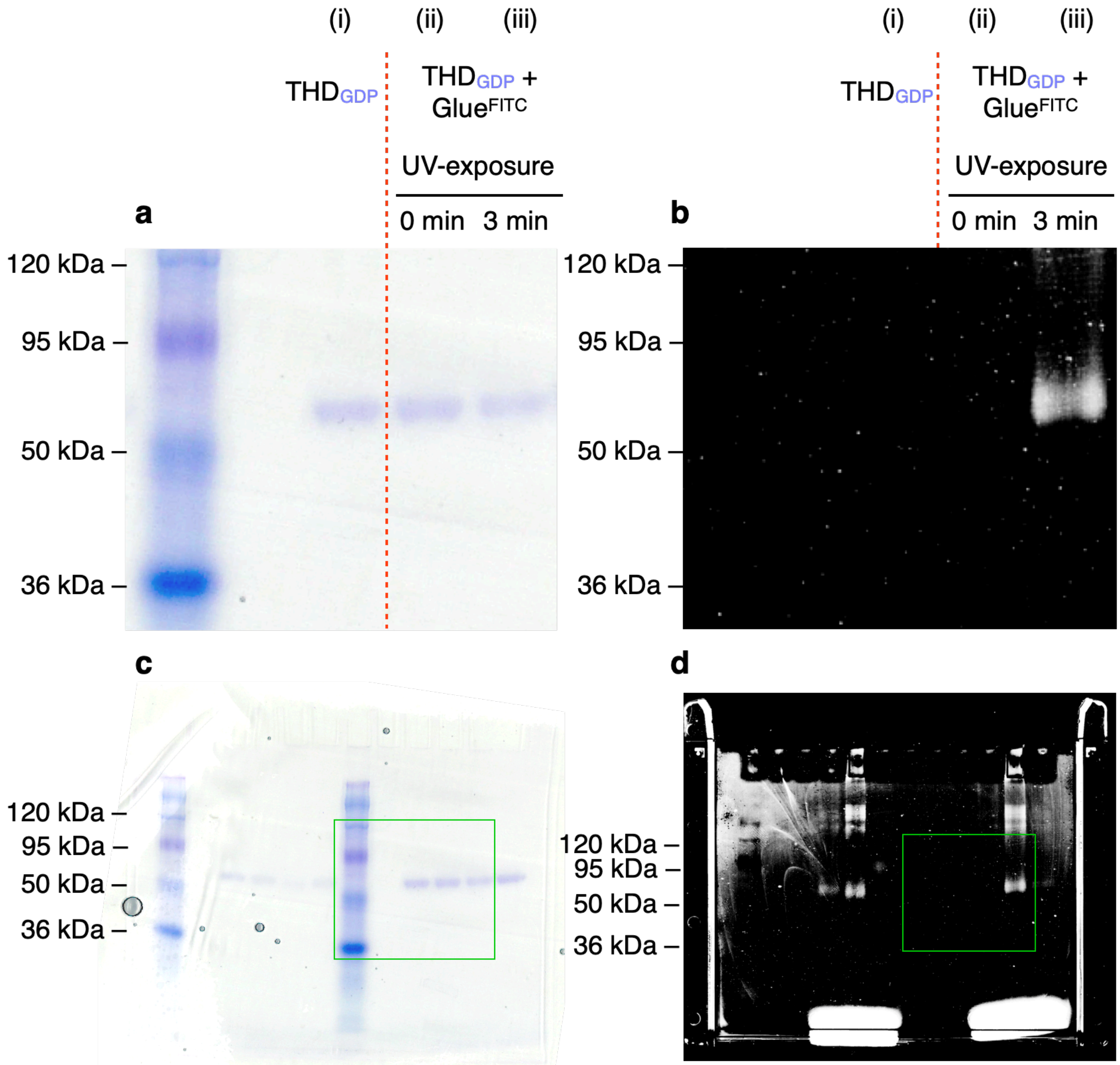

Supplementary Fig. 17. **a**, **b**, Sodium dodecyl sulfate polyacrylamide gel electrophoresis (SDS–PAGE) analysis of THD$_{GDP}$ (0.15 mg ml^{-1}; i) and a mixture of THD$_{GDP}$ (0.15 mg ml^{-1}) and Glue^{FITC} (10 μM) in PIPES buffer (20 mM PIPES, pH 6.8) before (ii) and after (iii) irradiation with 300-nm light for 3 min. The samples were visualized by staining with Coomassie Brilliant Blue (**a**) and by observing the fluorescence emission of FITC (λ_{ext} = 488 nm, λ_{obs} = 526 nm; **b**). **c**, **d**, Uncropped version of gel images of **a** (**c**) and **b** (**d**), where green squares indicate cropped area.

2-10. AFM observation of $^{CL}NC_{GTP/GTP*}$

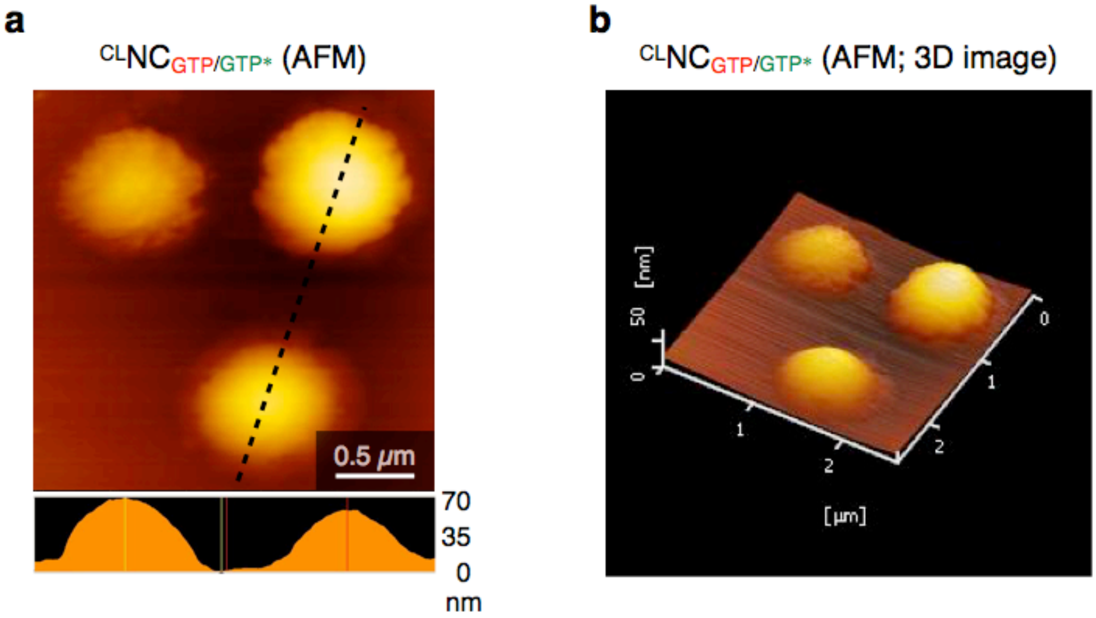

Supplementary Fig. 18. **a**, **b**, 2D (**a**) and 3D (**b**) AFM images of $^{CL}NC_{GTP/GTP*}$ (13 μg ml^{-1}).

2-11. Stability of $NC_{GTP/GTP*}$ and $^{CL}NC_{GTP/GTP*}$ in the presence of BSA

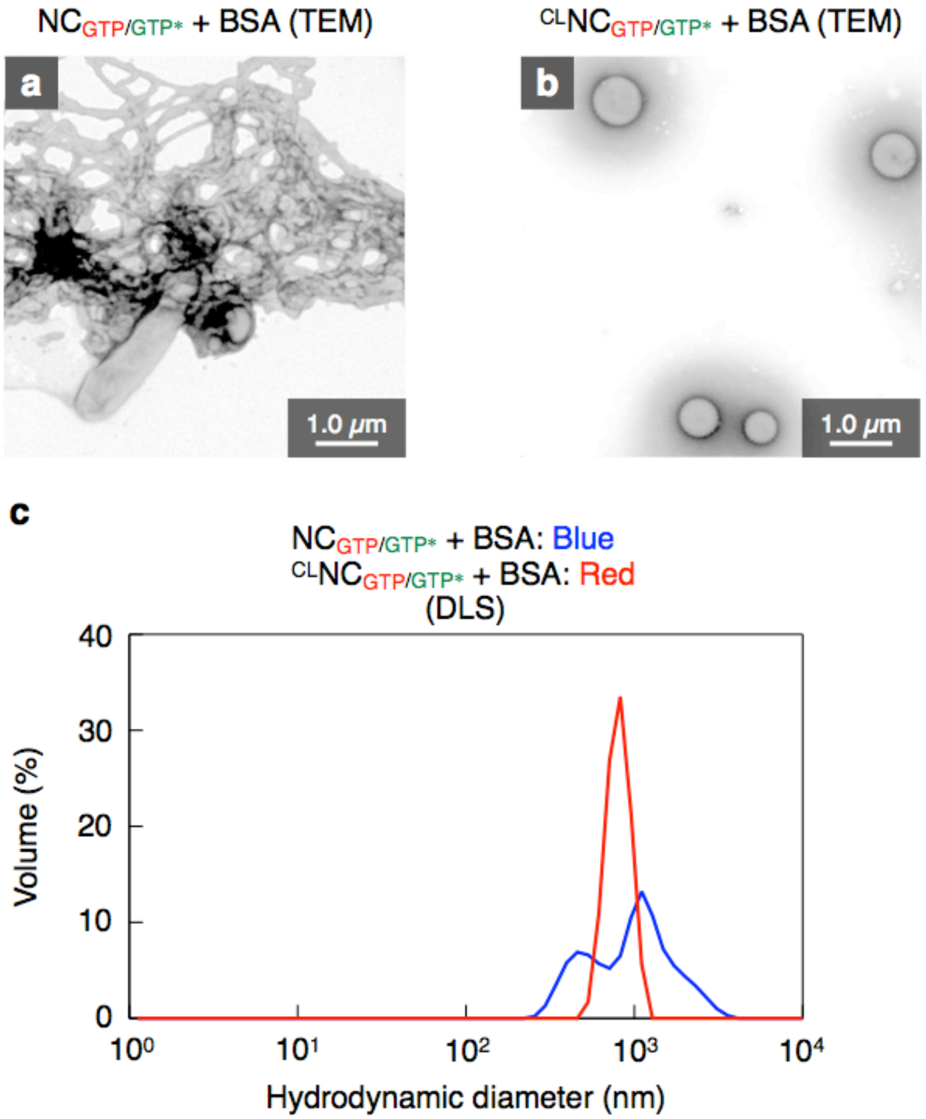

Supplementary Fig. 19. **a**, **b**, TEM images of a solution of $NC_{GTP/GTP*}$ (13 μg ml^{-1}; **a**) and $^{CL}NC_{GTP/GTP*}$ (13 μg ml^{-1}; **b**) in PIPES buffer (14 mM PIPES, 1 mM $MgCl_2$, and 0.2 mM GTP*, pH 6.8) after an incubation with BSA (0.1 mg ml^{-1}) at 37 °C for 30 min. **c**, DLS profiles of $NC_{GTP/GTP*}$ (blue) and $^{CL}NC_{GTP/GTP*}$ (red) after the incubation with BSA.

2-12. Stability of $NC_{GTP/GTP*}$ and $^{CL}NC_{GTP/GTP*}$ in the presence of FBS

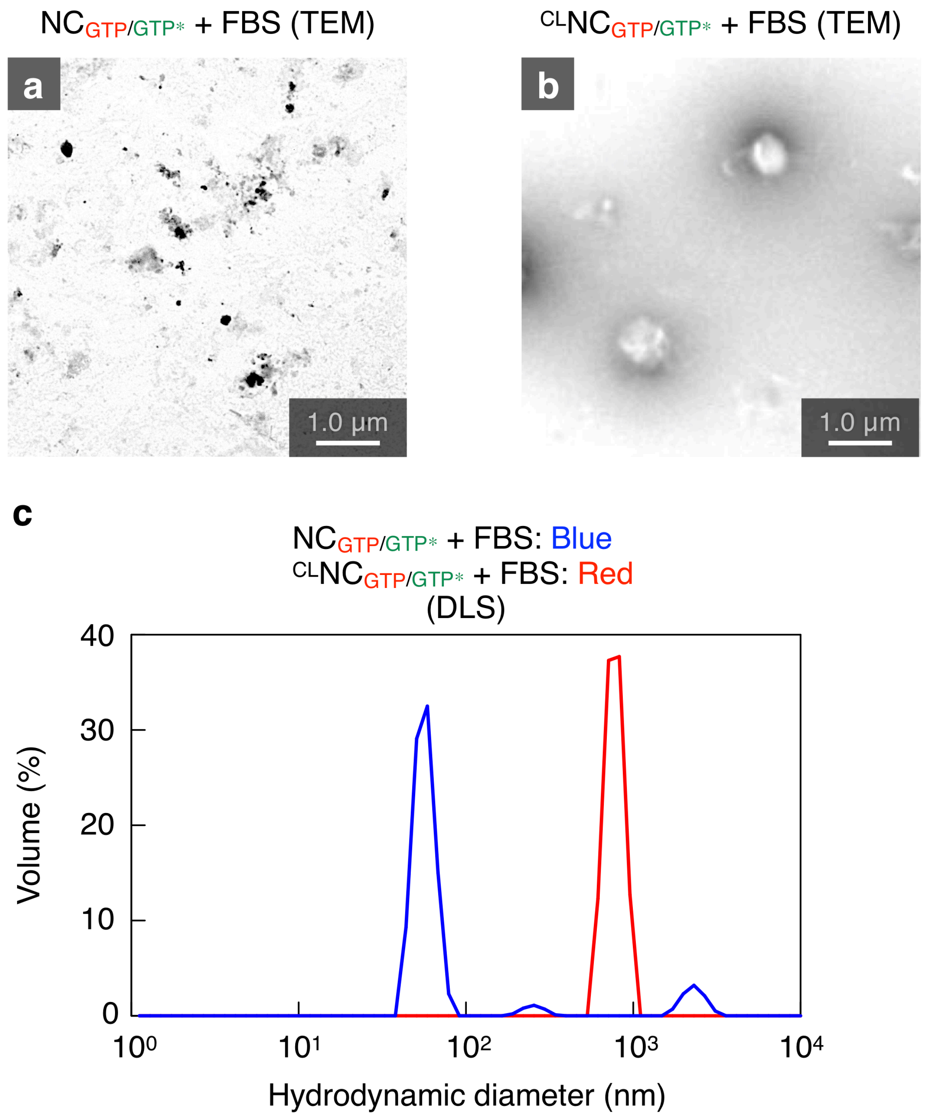

Supplementary Fig. 20. **a**, **b**, TEM images of $NC_{GTP/GTP*}$ (13 μg ml^{-1}; **a**) and $^{CL}NC_{GTP/GTP*}$ (13 μg ml^{-1}; **b**) after an incubation with FBS (0.01%) at 37 °C for 15 min in PIPES buffer (14 mM PIPES, 1 mM $MgCl_2$, and 0.2 mM GTP*, pH 6.8). **c**, DLS profiles of $NC_{GTP/GTP*}$ (blue) and $^{CL}NC_{GTP/GTP*}$ (red) after the incubation with FBS.

2-13. Formation of fluorescent $NC_{GTP/GTP*}$ and $^{CL}NC_{GTP/GTP*}$ using $Glue^{FITC}$

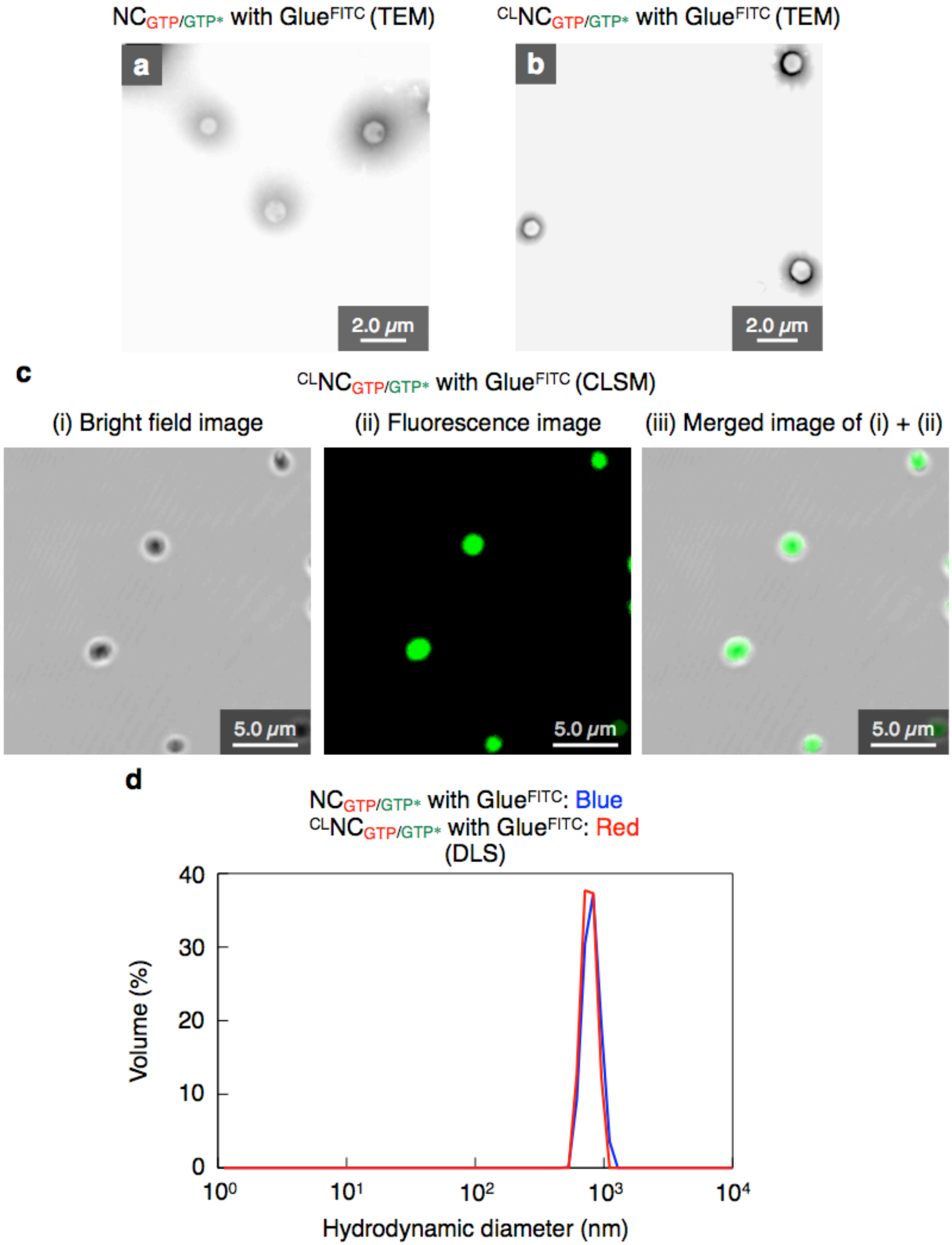

Supplementary Fig. 21. **a**, **b**, TEM images of $NC_{GTP/GTP*}$ (13 μg ml^{-1}; **a**) and $^{CL}NC_{GTP/GTP*}$ (13 μg ml^{-1}; **b**) prepared using $Glue^{FITC}$ (100 μM) in PIPES buffer (14 mM PIPES, 1 mM $MgCl_2$, and 0.2 mM GTP*, pH 6.8). **c**, CLSM observation of $^{CL}NC_{GTP/GTP*}$ prepared using $Glue^{FITC}$ (0.5 μg ml^{-1}). (i) Bright field image, (ii) fluorescence image (λ_{ext} = 488 nm, λ_{obs} = 505–565 nm), and (iii) a merged image of (i) and (ii). **d**, DLS profiles of $NC_{GTP/GTP*}$ (blue curve) and $^{CL}NC_{GTP/GTP*}$ (red curve) in PIPES buffer.

2-14. MD simulation of the folding of Glue$^{CO_2^-}$ in aqueous solution

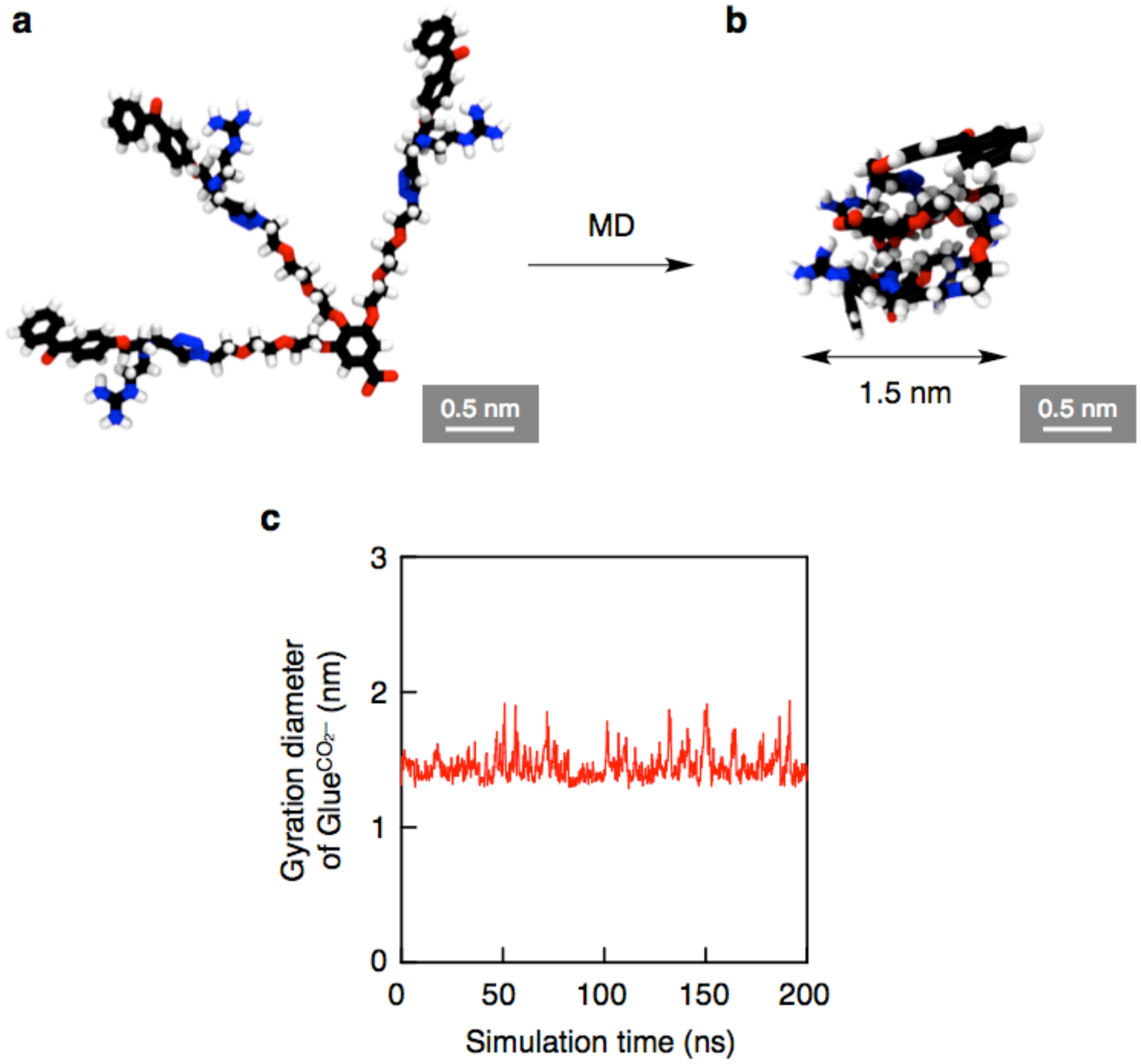

Supplementary Fig. 22. **a**, **b**, Snapshots of Glue$^{CO_2^-}$ before (**a**) and after (**b**) the MD simulation. Nitrogen, oxygen, carbon, and hydrogen atoms of Glue$^{CO_2^-}$ are colored in blue, red, grey, and white, respectively. Water molecules and chloride anions are not shown explicitly. **c**, Gyration diameter of Glue$^{CO_2^-}$ calculated along 200 ns by MD simulation.

2-15. MD simulation of the interaction of Glue$^{CO_2^-}$ with THD_{GTP*}

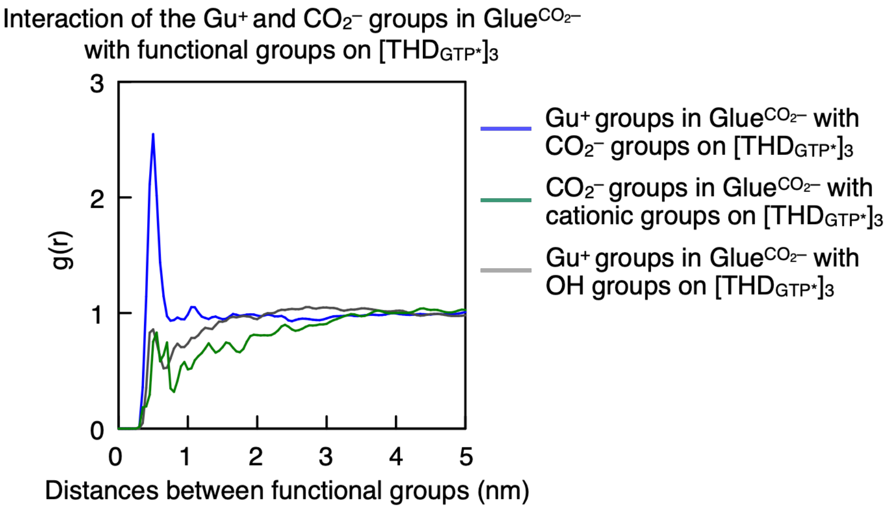

Supplementary Fig. 23. Radial distribution functions g(r) of the Gu^+ groups in Glue$^{CO_2^-}$ with CO_2^- groups (blue) and nonionic hydroxyl groups (grey) on $[THD_{GTP*}]_3$, and the CO_2^- group at the focal core of Glue$^{CO_2^-}$ with cationic groups on $[THD_{GTP*}]_3$ (green).

2-16. $NS_{GTP/GTP*}$ treated with $Glue^{CO_2Me}$

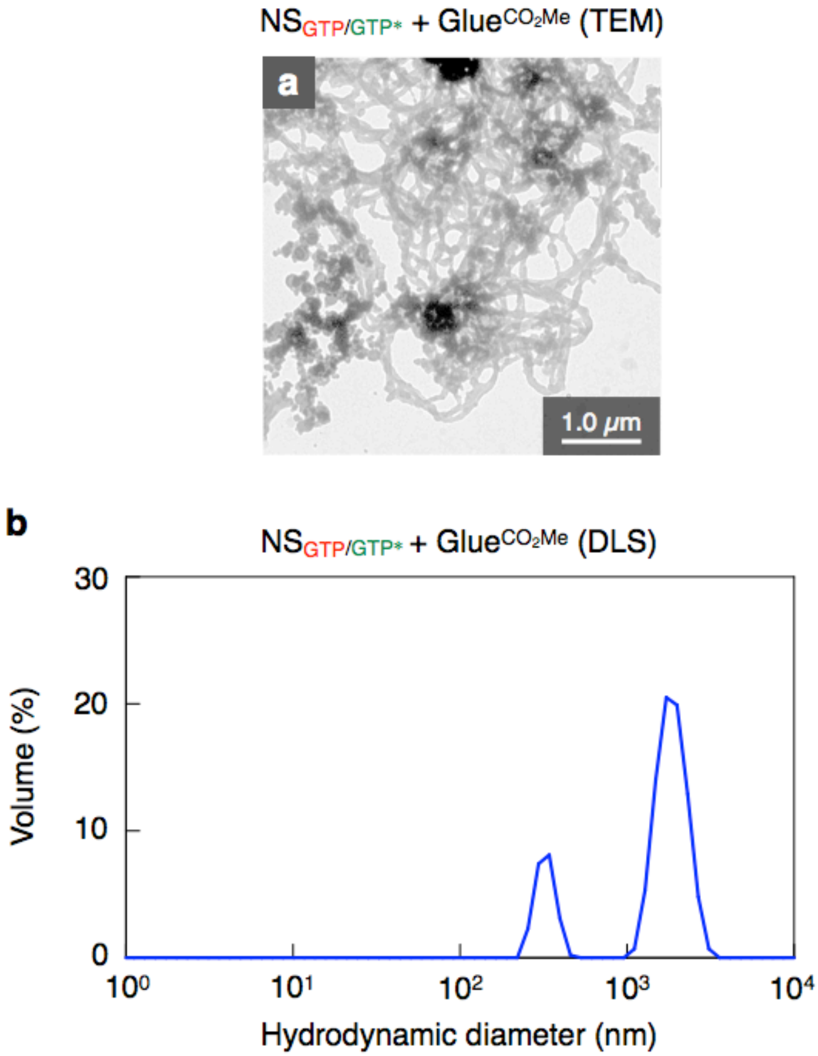

Supplementary Fig. 24. a, **b**, TEM image (**a**) and DLS profile (**b**) of $NS_{GTP/GTP*}$ (13 μg ml^{-1}) in PIPES buffer (14 mM PIPES, 1 mM $MgCl_2$, and 0.2 mM GTP*, pH 6.8) after an incubation with $Glue^{CO_2Me}$ (100 μM) at 37 °C for 30 min.

2-17. GTPase activity of THD depending on hybridization with GTP*

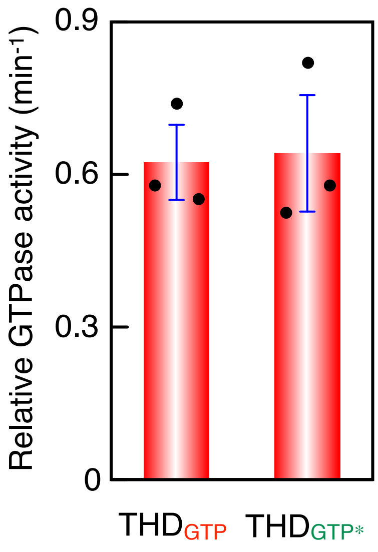

Supplementary Fig. 25. GTPase activities of THD_{GTP} (left) and THD_{GTP*} (right) in PIPES buffer (14 mM PIPES, 1 mM $MgCl_2$, pH 6.8). Red bars represent mean values ± SD from three different samples.

2-18. $^{CL}NC_{GTP/GTP*}$ treated with triphosphates

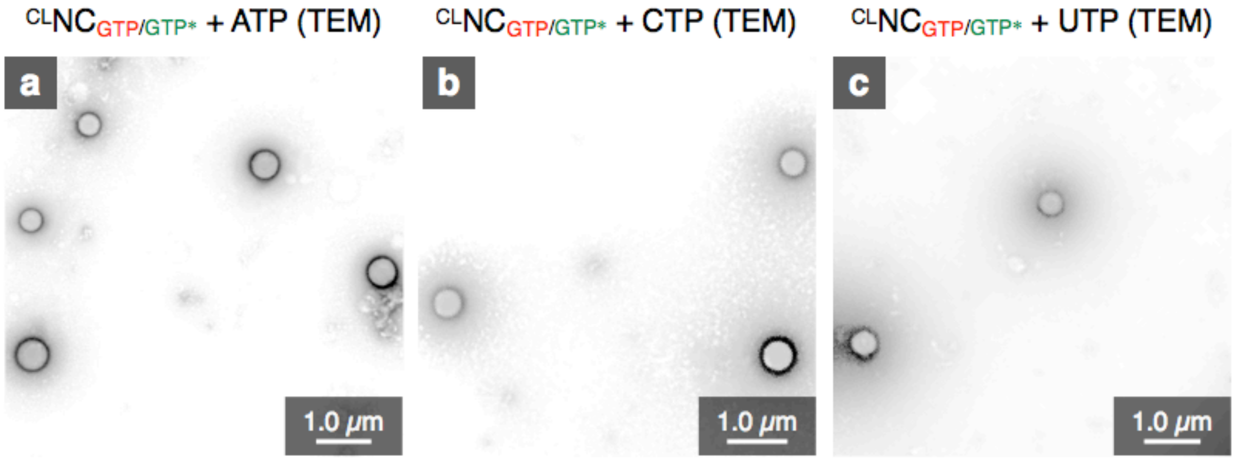

Supplementary Fig. 26. **a**–**c**, TEM images of $^{CL}NC_{GTP/GTP*}$ (9 μg ml^{-1}) after an incubation with 0.5 mM of ATP (**a**), CTP (**b**), or UTP (**c**) at 37 °C for 100 min in PIPES buffer (13 mM PIPES, 0.7 mM $MgCl_2$, and 0.1 mM GTP*, pH 6.8).

2-19. $^{CL}NC_{GTP/GTP*}$ without addition of GTP

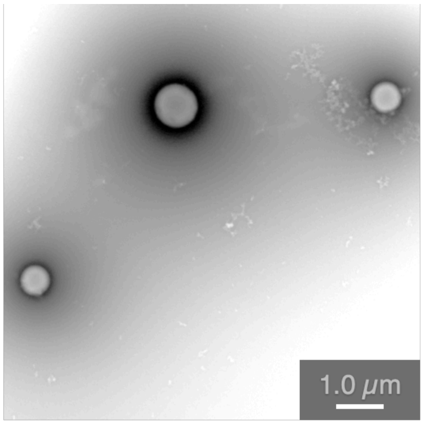

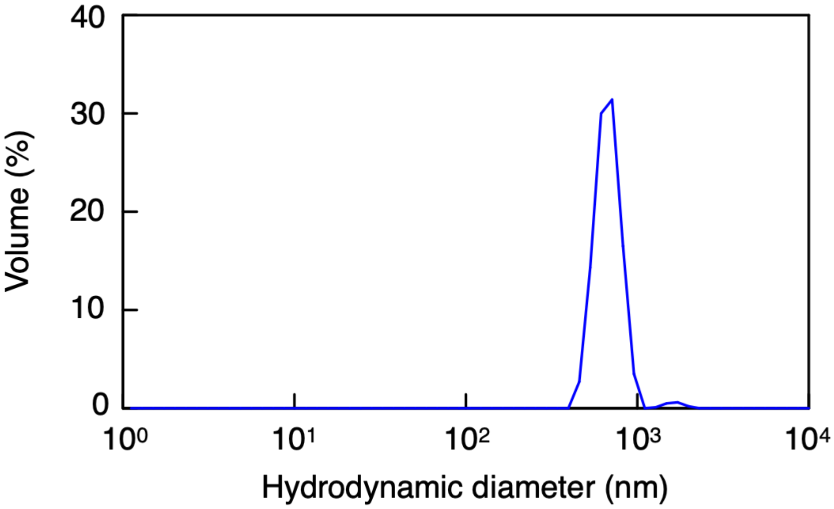

Supplementary Fig. 27. **a**, **b**, TEM image (**a**) and DLS profile (**b**) of $^{CL}NC_{GTP/GTP*}$ (9 μg ml^{-1}) after an incubation at 37 °C for 100 min in PIPES buffer (13 mM PIPES, 0.7 mM $MgCl_2$, and 0.1 mM GTP*, pH 6.8).

2-20. Encapsulation of NP_{Au} into $^{CL}NC_{GTP/GTP*}$

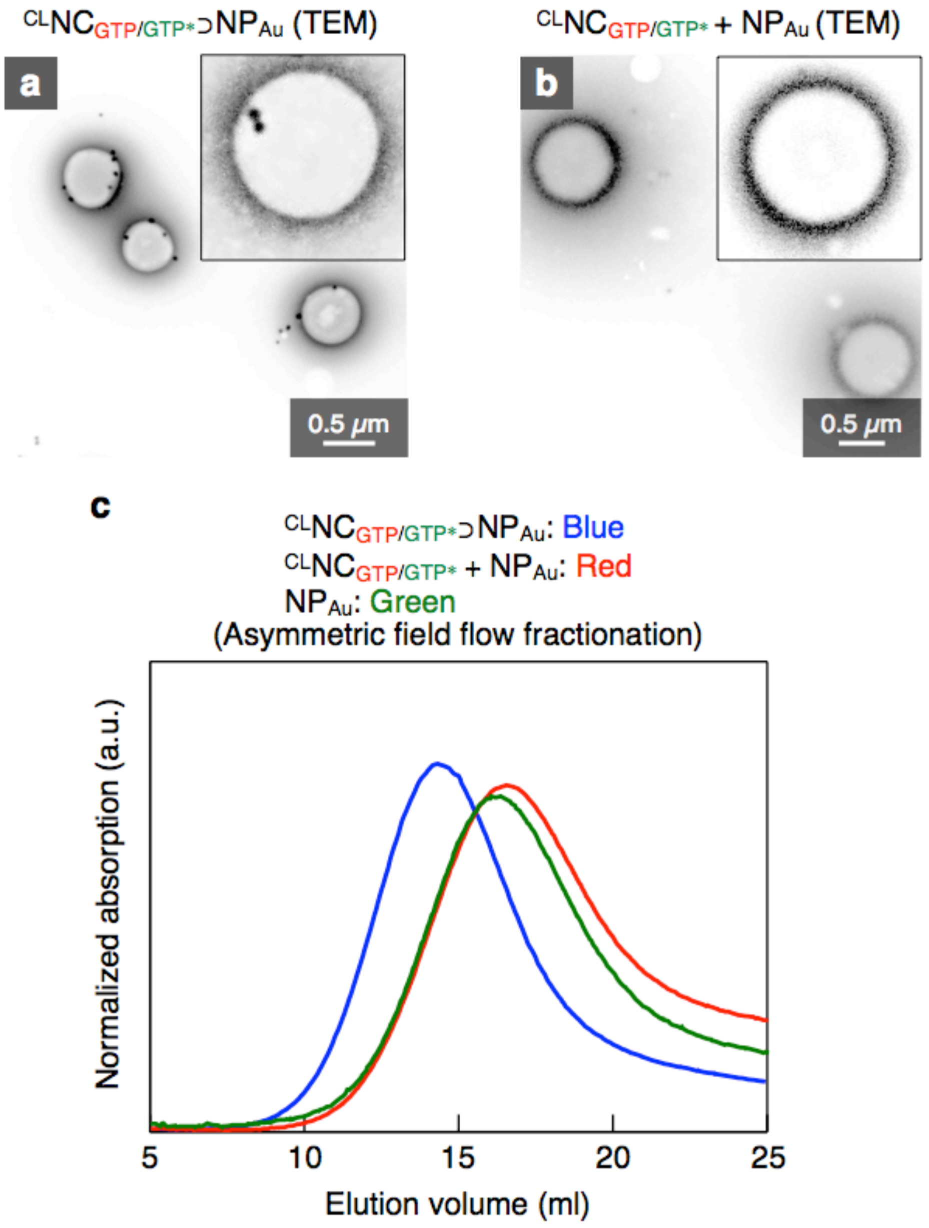

Supplementary Fig. 28. a, b, TEM images of $^{CL}NC_{GTP/GTP*} \supset NP_{Au}$ ([$^{CL}NC_{GTP/GTP*}$] = 13 μg ml^{-1}, [NP_{Au}] = 13 pM; **a**) and a mixture of $^{CL}NC_{GTP/GTP*}$ (13 μg ml^{-1}) and NP_{Au} (13 pM; **b**). **c,** Elution profiles of $^{CL}NC_{GTP/GTP*} \supset NP_{Au}$ (blue), the mixture of $^{CL}NC_{GTP/GTP*}$ and NP_{Au} (red), and NP_{Au} (green) obtained using asymmetric field flow fractionation.

2-21. Encapsulation of DOX into $^{CL}NC_{GTP/GTP*}$

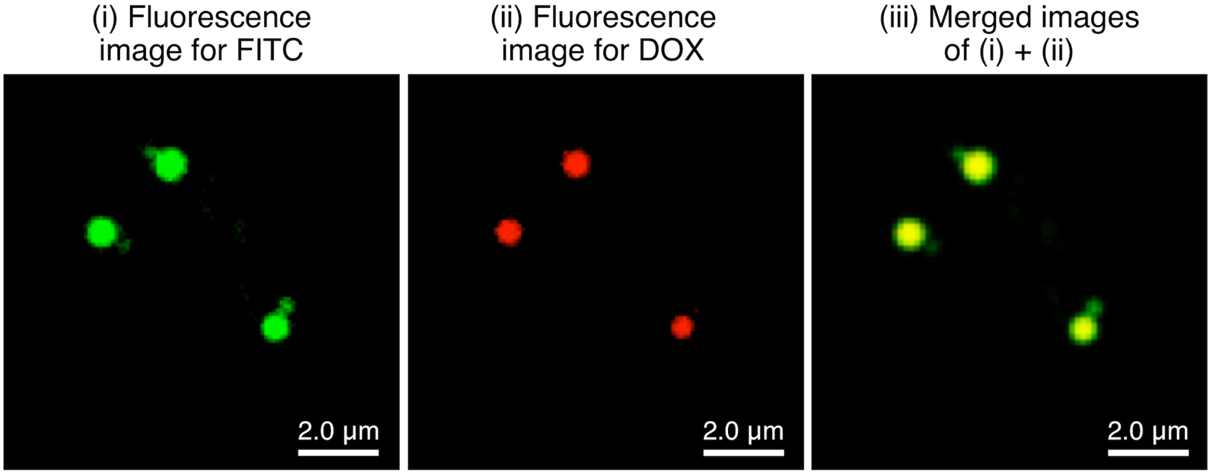

Supplementary Fig. 29. CLSM observations of FITC-labelled $^{CL}NC_{GTP/GTP*}\supset$DOX. (i) λ_{ext} = 488 nm, λ_{obs} = 505–520 nm for FITC, (ii) λ_{ext} = 488 nm, λ_{obs} = 570–645 nm for DOX, and (iii) a merged image of (i) and (ii).

2-22. Uptake of FITC-labelled THD_{GDP} into Hep3B cells

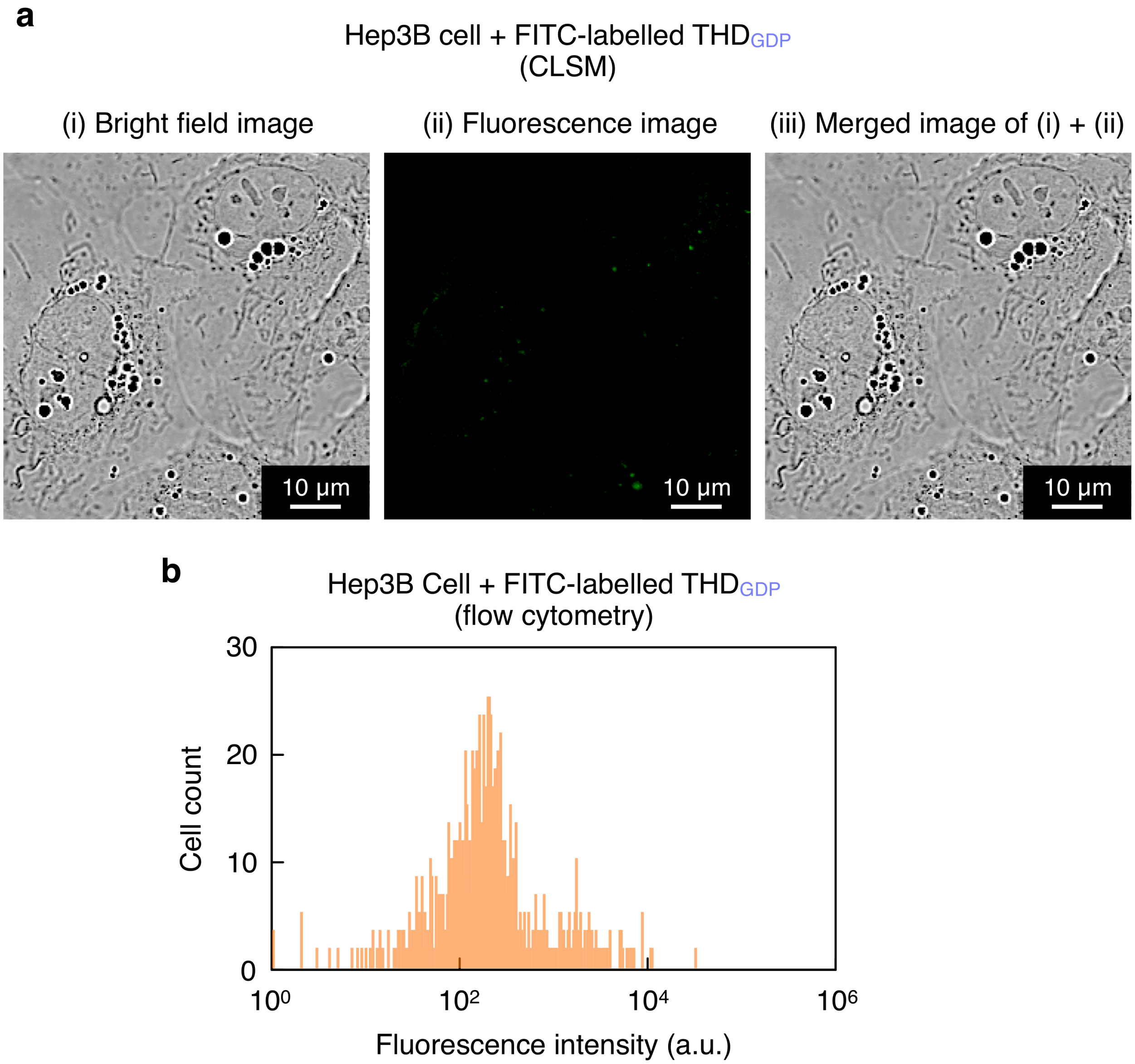

Supplementary Fig. 30. **a**, CLSM images of Hep3B cells after 2.5-h incubation with FITC-labelled THD_{GDP} (0.5 μg ml^{-1}) at 37 °C in EMEM and rinsing with D-PBS, followed by 1.5-h incubation in EMEM (10% FBS). (i) Bright field image, (ii) fluorescence image (λ_{ext} = 488 nm, λ_{obs} = 505–565 nm), and (iii) a merged image of (i) and (ii). **b**, Flow cytometry analysis of the Hep3B cell sample (n = 630).

2-23. Uptake of FITC-labelled $NS_{GTP/GTP*}$ into Hep3B cells

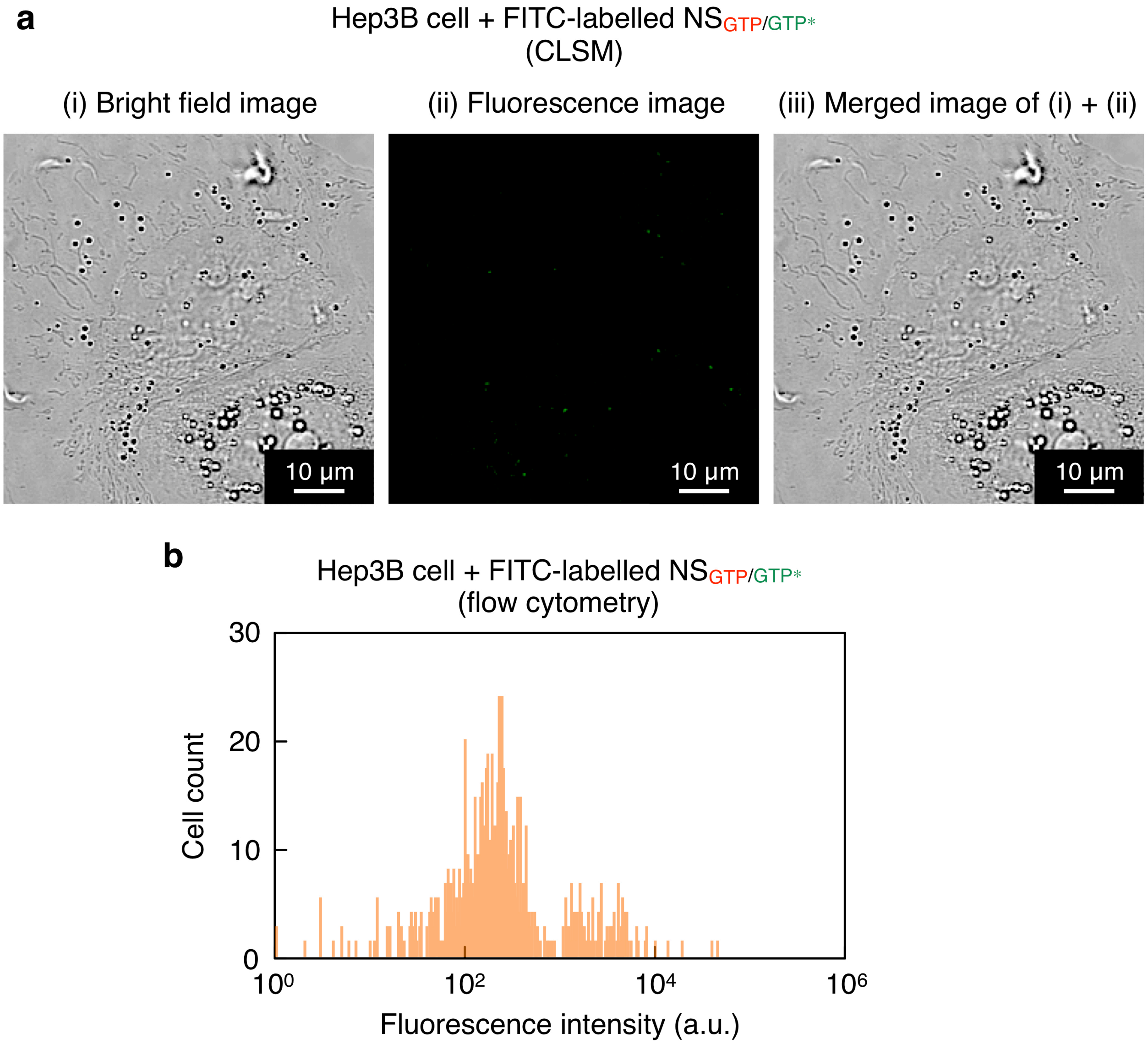

Supplementary Fig. 31. **a**, CLSM images of Hep3B cells after 2.5-h incubation with FITC-labelled $NS_{GTP/GTP*}$ (0.5 μg ml^{-1}) at 37 °C in EMEM and rinsing with D-PBS, followed by 1.5-h incubation in EMEM (10% FBS). (i) Bright field image, (ii) fluorescence image (λ_{ext} = 488 nm, λ_{obs} = 505–565 nm), and (iii) a merged image of (i) and (ii). **b**, Flow cytometry analysis of the Hep3B cell sample (n = 630).

2-24. Uptake of FITC-labelled $^{CL}NC_{GTP/GTP*}$ into Hep3B cells treated with an endocytosis inhibitor

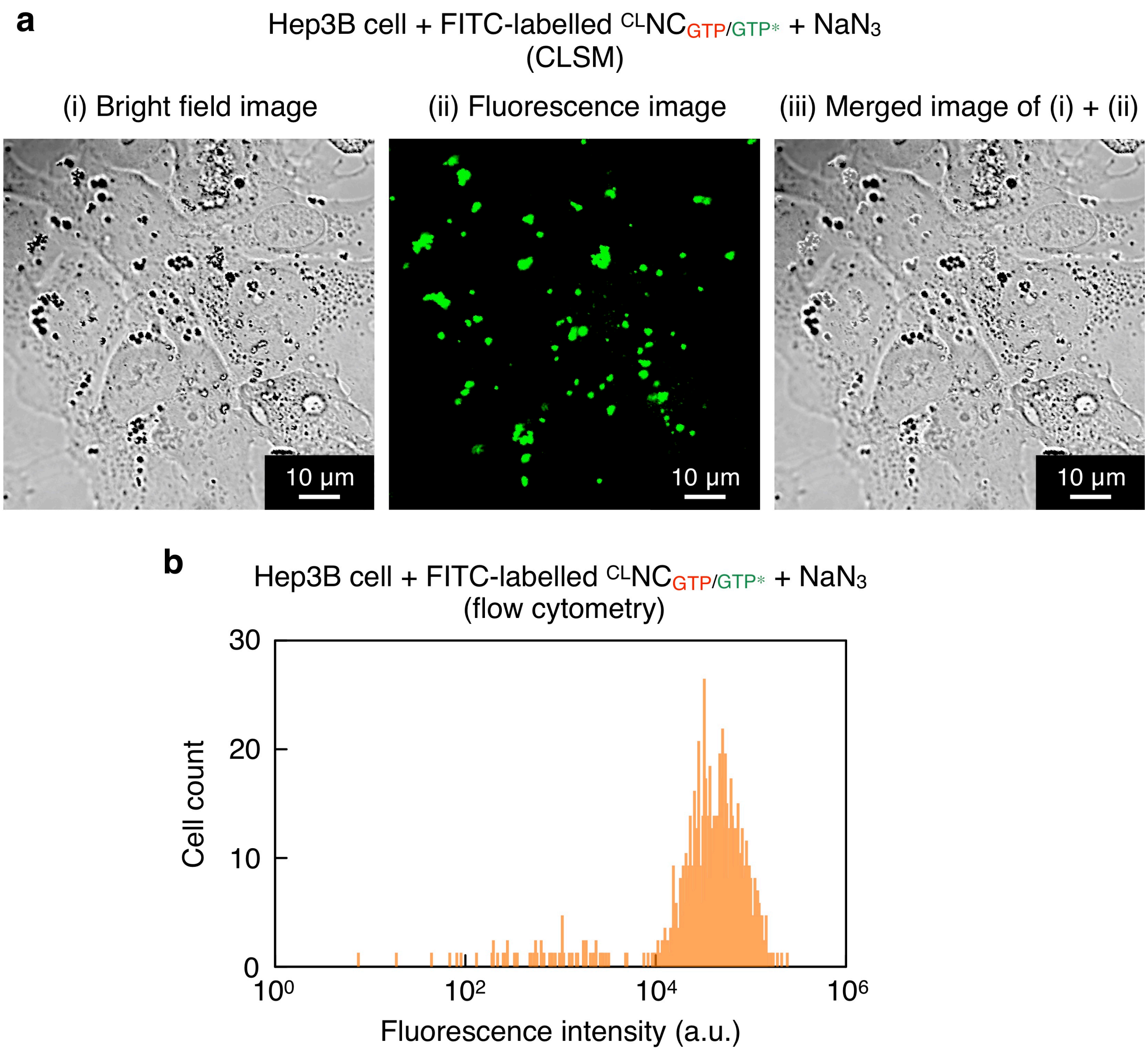

Supplementary Fig. 32. **a**, CLSM images of Hep3B cells after 2.5-h incubation with FITC-labelled $^{CL}NC_{GTP/GTP*}$ (0.5 μg ml^{-1}) in the presence of 5 mM of NaN_3 (endocytosis inhibitor) at 37 °C in EMEM and rinsing with D-PBS, followed by 1.5-h incubation in EMEM (10% FBS). (i) Bright field image, (ii) fluorescence image (λ_{ext} = 488 nm, λ_{obs} = 505–565 nm), and (iii) a merged image of (i) and (ii). **b**, Flow cytometry analysis of the Hep3B cell sample (n = 700).

2-25. Dose dependency on cytotoxicity of $^{CL}NC_{GTP/GTP*} \supset$ DOX

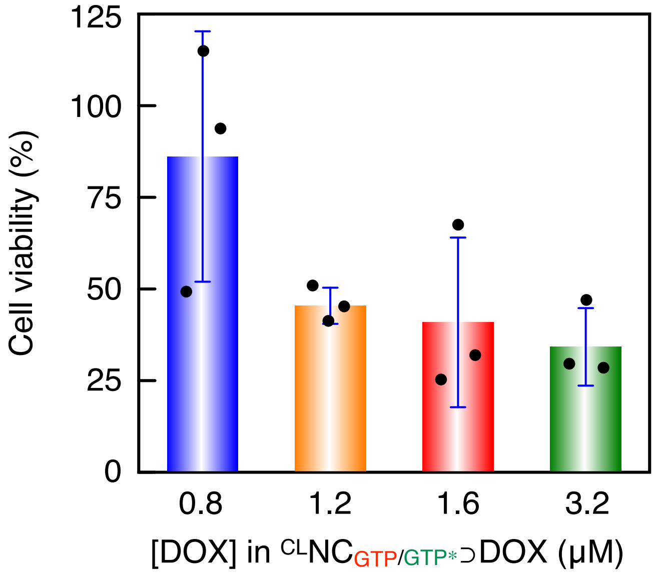

Supplementary Fig. 33. Normalized viabilities of Hep3B cells determined using Cell Counting Kit-8. Hep3B cells were treated with four different concentrations of $^{CL}NC_{GTP/GTP*} \supset$ DOX ([$^{CL}NC_{GTP/GTP*}$] = 1.0 μg ml^{-1}, [DOX] = 0.8 μM; blue), ([$^{CL}NC_{GTP/GTP*}$] = 1.6 μg ml^{-1}, [DOX] = 1.2 μM; orange), ([$^{CL}NC_{GTP/GTP*}$] = 2.1 μg ml^{-1}, [DOX] = 1.6 μM; red), and ([$^{CL}NC_{GTP/GTP*}$] = 4.2 μg ml^{-1}, [DOX] = 3.2 μM; green) for 2.5 h in EMEM, rinsed with D-PBS, and incubated in EMEM (10% FBS) for 21.5 h. Bars represent mean values ± SD from three different samples.

2-26. Incubation time dependency on cytotoxicity of $^{CL}NC_{GTP/GTP*} \supset DOX$

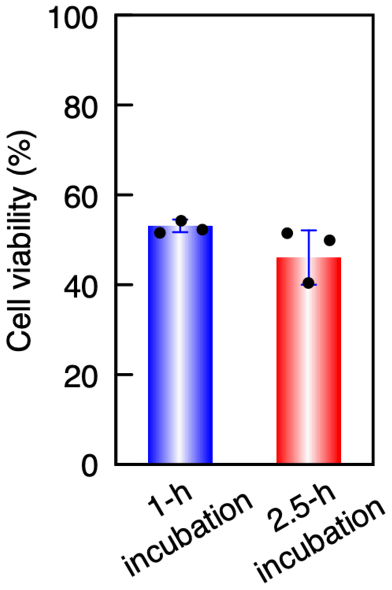

Supplementary Fig. 34. Normalized viabilities of Hep3B cell determined using Cell Counting Kit-8. Hep3B cells were incubated in EMEM containing $^{CL}NC_{GTP/GTP*} \supset DOX$ ($[^{CL}NC_{GTP/GTP*}]$ = 1.6 μg ml^{-1}, [DOX] = 1.2 μM) for 1 h (blue) or 2.5 h (red), rinsed with D-PBS, and further incubated in EMEM (10% FBS) for 21.5 h. Bars represent mean values ± SD from three different samples.

2-27. Cell line dependency on cytotoxicity of $^{CL}NC_{GTP/GTP*} \supset DOX$

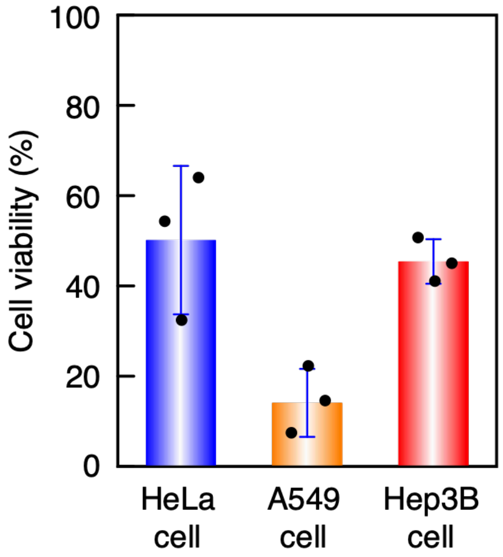

Supplementary Fig. 35. Normalized viabilities of HeLa cell (blue), A549 cell (orange), and Hep3B cell (red) determined using Cell Counting Kit-8. Cells were incubated in EMEM containing $^{CL}NC_{GTP/GTP*} \supset DOX$ ([$^{CL}NC_{GTP/GTP*}$] = 1.6 μg ml^{-1}, [DOX] = 1.2 μM) for 2.5 h, rinsed with D-PBS, and further incubated in EMEM (10% FBS) for 21.5 h. Bars represent mean values ± SD from three different samples.

2-28. Effects of encapsulation of DOX in $^{CL}NC_{GTP/GTP*}$ ⊃DOX for cytotoxicity test

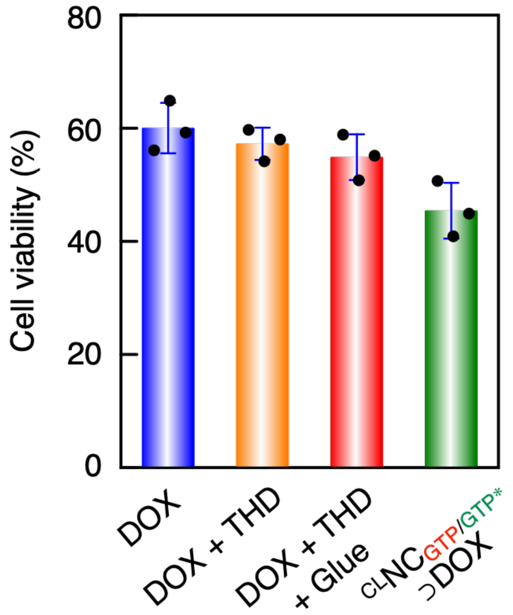

Supplementary Fig. 36. Normalized viabilities of Hep3B cells determined using Cell Counting Kit-8. Hep3B cells were incubated in EMEM for 2.5 h containing DOX (1.2 μM; blue), a mixture of DOX and THD_{GDP} ([DOX] = 1.2 μM, [THD_{GDP}] = 1.6 μg; orange), a mixture of DOX, THD_{GDP}, and $Glue^{CO_2-}$ ([DOX] = 1.2 μM, [THD_{GDP}] = 1.6 μg, [$Glue^{CO_2-}$] = 12.3 μM; red), and $^{CL}NC_{GTP/GTP*}$ ⊃DOX ([$^{CL}NC_{GTP/GTP*}$] = 1.6 μg ml^{-1}, [DOX] = 1.2 μM; green), rinsed with D-PBS, and further incubated in EMEM (10% FBS) for 21.5 h. Bars represent mean values ± SD from three different samples.

3. Supplementary References

S1. Fujisawa, T. et al. Small-angle X-ray scattering station at the SPring-8 RIKEN beamline. *J. Appl. Cryst.* **33**, 797–800 (2000).

S2. Garzoni, M., Okuro, K., Ishii, N., Aida, T. & Pavan, G. M. Structure and shape effects of molecular glue on supramolecular tubulin assemblies. *ACS Nano* **8**, 904–914 (2014).

S3. Case, D. A. et al. The amber biomolecular simulation programs. *J. Comput. Chem.* **26**, 1668–1688 (2005).

S4. Hess, B., Kutzner, C., van der Spoel, D. & Lindahl, E. GROMACS 4: algorithms for highly efficient, load-balanced, and scalable molecular simulation. *J. Chem. Theory Comput.* **4**, 435–447 (2008).

S5. Humphrey, W., Dalke, A. & Schulten, K. VMD: visual molecular dynamics. *J. Mol. Graph.* **14**, 33–38 (1996).

S6. Uchida, N. et al. Photoclickable dendritic molecular glue: noncovalent-to-covalent photochemical transformation of protein hybrids. *J. Am. Chem. Soc.* **135**, 4684–4687 (2013).

S7. Amaral, S. P., Fernandez-Villamarin, M., Correa, J., Riguera, R. & Fernandez-Megia, E. Efficient multigram synthesis of the repeating unit of gallic acid-triethylene glycol dendrimers. *Org. Lett.* **13**, 4522–4525 (2011).

S8. Mukherjee, G., Patra, N., Barua, P. & Jayaram, B. A fast empirical GAFF compatible partial atomic charge assignment scheme for modeling interactions of small molecules with biomolecular targets. *J. Comput. Chem.* **32**, 893–907 (2011).

S9. Jorgensen, W. L., Chandrasekhar, J., Madura, J. D., Impey, R. W. & Klein, M. L. Comparison of simple potential functions for simulating liquid water. *J. Chem. Phys.* **79**, 926–935 (1983).

S10. Alushin, G. M. et al. High-resolution microtubule structures reveal the structural transitions in *αβ*-tubulin upon GTP hydrolysis. *Cell* **157**, 1117–1129 (2014).

S11. Nogales, E., Wolf, S. G. & Downing, K. H. Structure of the *αβ* tubulin dimer by electron crystallography. *Nature* **391**, 199–203 (1998).

S12. Hornak, V. et al. Comparison of multiple AMBER force fields and development of improved protein backbone parameters. *Proteins* **65**, 712–725 (2006).

S13. Darden, T., York, D. & Pedersen, L. Particle mesh Ewald: an Nlog(N) method for Ewald sums in large systems. *J. Chem. Phys.* **98**, 10089–10092 (1993).

S14. Bussi, G., Donadio, D. & Parrinello, M. Canonical sampling through velocity-rescaling. *J. Chem. Phys.* **126**, 014101 (2007).

S15. Berendsen, H. J. C., Postma, J. P. M., van Gunsteren, W. F., DiNola, A. & Haak, J. R. Molecular dynamics with coupling to an external bath. *J. Chem. Phys.* **81**, 3684–3690 (1984).

S16. Eisenhaber, F., Lijnzaad, P., Argos, P., Sander, C. & Scharf, M. The double cubic lattice method: efficient approaches to numerical integration of surface area and volume and to dot surface contouring of molecular assemblies. *J. Comput. Chem.* **16**, 273–284 (1995).

S17. Baker, N. A., Sept, D., Joseph, S., Holst, M. J. & McCammon, J. A. Electrostatics of nanosystems: application to microtubules and the ribosome. *Proc. Natl. Acad. Sci. USA* **98**, 10037–10041 (2001).

S18. Yamaguchi, D., Miyamoto, N., Koizumi, S., Nakato, T. & Hashimoto, T. Hierarchical structure of niobate nanosheets in aqueous solutions. *Appl. Cryst.* **40***,* s101– s105 (2007).
