# Supplementary material for: Reconstitution of microtubule into GTP-responsive nanocapsules
Source: Nat Commun. 2022 Sep 15;13:5424. doi: 10.1038/s41467-022-33156-5 (PMC9477877; doi:10.1038/s41467-022-33156-5)
Supplement: Supplementary file 3 — Reporting Summary [file 41467_2022_33156_MOESM3_ESM.pdf]

nature portfolio

Corresponding author(s): Takuzo Aida
Last updated by author(s): Aug 7, 2022

Reporting Summary

Nature Portfolio wishes to improve the reproducibility of the work that we publish. This form provides structure for consistency and transparency in reporting. For further information on Nature Portfolio policies, see our Editorial Policies and the Editorial Policy Checklist.

Statistics

For all statistical analyses, confirm that the following items are present in the figure legend, table legend, main text, or Methods section.

n/a	Confirmed	
☐	☒	The exact sample size (*n*) for each experimental group/condition, given as a discrete number and unit of measurement
☐	☒	A statement on whether measurements were taken from distinct samples or whether the same sample was measured repeatedly
☐	☒	The statistical test(s) used AND whether they are one- or two-sided<br>*Only common tests should be described solely by name; describe more complex techniques in the Methods section.*
☐	☒	A description of all covariates tested
☐	☒	A description of any assumptions or corrections, such as tests of normality and adjustment for multiple comparisons
☐	☒	A full description of the statistical parameters including central tendency (e.g. means) or other basic estimates (e.g. regression coefficient) AND variation (e.g. standard deviation) or associated estimates of uncertainty (e.g. confidence intervals)
☐	☒	For null hypothesis testing, the test statistic (e.g. *F*, *t*, *r*) with confidence intervals, effect sizes, degrees of freedom and *P* value noted<br>*Give P values as exact values whenever suitable.*
☒	☐	For Bayesian analysis, information on the choice of priors and Markov chain Monte Carlo settings
☒	☐	For hierarchical and complex designs, identification of the appropriate level for tests and full reporting of outcomes
☒	☐	Estimates of effect sizes (e.g. Cohen's *d*, Pearson's *r*), indicating how they were calculated

Our web collection on statistics for biologists contains articles on many of the points above.

Software and code

Policy information about availability of computer code

Data collection	The MT molecular model was built from PDB (code: 3J6E and 1TUB) using AmberTools 20 and the GAFF force field as a basis. All simulations were run with GROMACS 2020.5. All images were created with the Visual Molecular Dynamics (VMD) package. See Supplementary Information for additional details.
Data analysis	BD Accuri C6 software was used for analysis of flow cytometory data. See Supplementary Information.

For manuscripts utilizing custom algorithms or software that are central to the research but not yet described in published literature, software must be made available to editors and reviewers. We strongly encourage code deposition in a community repository (e.g. GitHub). See the Nature Portfolio guidelines for submitting code & software for further information.

Data

Policy information about availability of data

All manuscripts must include a data availability statement. This statement should provide the following information, where applicable:

- Accession codes, unique identifiers, or web links for publicly available datasets
- A description of any restrictions on data availability
- For clinical datasets or third party data, please ensure that the statement adheres to our policy

All the data corresponding to the findings of this study are provided in the article and Supplementary Information. 3D structures of tubulin protein for the MD simulation were obtained from Protein Data Bank (PDB) (PDB code: 3J6E, [https://www.rcsb.org/structure/3j6e] and PDB code: 1TUB, [https://www.rcsb.org/structure/1TUB]). Complete modeling data, structures and parameters used for, and extracted from simulations are available at https://github.com/GMPavanLab/TubulinNCs/ (this temporary link will be replaced with a definitive Zenodo link upon acceptance of the paper).

March 2021

Field-specific reporting

Please select the one below that is the best fit for your research. If you are not sure, read the appropriate sections before making your selection.

- [x] Life sciences
- [ ] Behavioural & social sciences
- [ ] Ecological, evolutionary & environmental sciences

For a reference copy of the document with all sections, see nature.com/documents/nr-reporting-summary-flat.pdf

Life sciences study design

All studies must disclose on these points even when the disclosure is negative.

Sample size	The sample size in the cell experiments in this study were determined on flow cytometry analysis according to our previous experience and other publications (ex. Front. Oncol. 2022, 12, 843742).
Data exclusions	No data was excluded.
Replication	All attempts (n = 3) regarding cell experiments at replication were successful.
Randomization	All samples were allocated randomly.
Blinding	Experiments were not blinded because the results of the experiments in this study are not influenced by preconceived notions, which don't require blind testings.

Reporting for specific materials, systems and methods

We require information from authors about some types of materials, experimental systems and methods used in many studies. Here, indicate whether each material, system or method listed is relevant to your study. If you are not sure if a list item applies to your research, read the appropriate section before selecting a response.

Materials & experimental systems

n/a	Involved in the study	
x		Antibodies
	x	Eukaryotic cell lines
x		Palaeontology and archaeology
x		Animals and other organisms
x		Human research participants
x		Clinical data
x		Dual use research of concern

Methods

n/a	Involved in the study	
x		ChIP-seq
	x	Flow cytometry
x		MRI-based neuroimaging

Eukaryotic cell lines

Policy information about cell lines

Cell line source(s)	Hep3B (ATCC, HB-8064TM), HeLa (JCRB9004), A549 (JCRB0076)
Authentication	Cells were not authenticated.
Mycoplasma contamination	No mycoplasma contamination was found.
Commonly misidentified lines (See ICLAC register)	None of the cell line used are listed in the ICLAC list.

Flow Cytometry

Plots

Confirm that:

- [x] The axis labels state the marker and fluorochrome used (e.g. CD4-FITC).
- [x] The axis scales are clearly visible. Include numbers along axes only for bottom left plot of group (a 'group' is an analysis of identical markers).
- [x] All plots are contour plots with outliers or pseudocolor plots.
- [x] A numerical value for number of cells or percentage (with statistics) is provided.

Methodology

Sample preparation	Hep3B cells were detached and subjected into Flow Cytometry immediately.
Instrument	BD model Accuri C6 flow cytometer
Software	BD Accuri C6 software
Cell population abundance	Samples were not purified.
Gating strategy	To distinguish "positive" and "negative" stained cells, the thresholds were set at 80,000 FSC and at 0 SSC using detectors FL1 for FITC and FL3 for DOX.

[x] Tick this box to confirm that a figure exemplifying the gating strategy is provided in the Supplementary Information.
